# Influence of different rootstock-interstock-scion combinations on mango (*Mangifera indica* L.) traits

**DOI:** 10.3389/fpls.2025.1625932

**Published:** 2025-09-03

**Authors:** Shikha Jain, Jai Prakash, Sanjay Kumar Singh, Chavlesh Kumar, Manish Srivastav, Kanhaiya Singh, Renu Pandey, Sandhya Sharma, Anshuman Singh, Ishu Kumari

**Affiliations:** ^1^ Division of Fruit and Horticultural Technology, Indian Agricultural Research Institute, New Delhi, India; ^2^ Division of Plant Physiology, Indian Agricultural Research Institute, New Delhi, India; ^3^ Indian Council of Agricultural Research (ICAR)-National Institute of Plant Biotechnology, New Delhi, India; ^4^ Division of Crop Improvement & Biotechnology, Indian Council of Agricultural Research (ICAR)-Central Institute of Subtropical Horticulture, Lucknow, India

**Keywords:** mango, rootstock, interstock, grafting, morpho-physio-chemical traits

## Abstract

Mango (*Mangifera indica* L.), a highly valued tropical fruit, faces challenges in productivity due to the use of non-descriptive rootstocks and large tree architecture. To address this, a field experiment was conducted at ICAR-IARI, New Delhi (2021-2024), using Olour as rootstock and scion, with three grafting combinations: without interstock, with Amrapali interstock, and with Mallika interstock. The study aimed to evaluate their effects on morpho-physio-chemical traits, leaf and soil nutrient content, and anatomical parameters. The results revealed significant differences in plant performance based on the treatment combinations. The Olour/Mallika/Olour combination showed the highest leaf width (3.71 cm),intercellular CO_2_ concentration (356.20μmole m^-2^ s^-1^), net photosynthetic rate (8.51 μmole m^-2^ s^-1^), leaf total soluble protein (4.34 mg/g FW), leaf total sugars (119.05 mg/g FW), total chlorophyll (4.04 mg/g FW), total carotenoid (0.22 mg/g FW) and stomatal density (746.00 mm^-2^) and lowest apical bud phenols (1014.31 mg/100 g) and leaf proline content (0.36 µg g^-1^ FW). Conversely, the Olour/Amrapali/Olour combination exhibited lowest rootstock girth(7.11 mm), scion girth (4.32 mm), leaf fresh weight (1.26 g), leaf dry weight (0.40 g), leaf net photosynthesis (3.50 μmole m^-2^ s^-1^), leaf total soluble protein (1.25 mg/g FW), total chlorophyll (1.65 mg/g FW), total carotenoid (0.13 mg/g FW) and stomatal density (380.75 mm^-2^) and demonstrated higher proline (1.06 µg g^-1^ FW) and apical bud phenols (3067.53 mg/100 g)indicating dwarfing potential. Among the single-graft combinations, Amrapali/Olour exhibited moderate vigour and nutrient content, while the Mallika/Olour combination maintained high stomatal conductance and favourable growth traits. These findings confirm that both interstock and direct scion-rootstock combinations significantly influence plant physiology and nutrient dynamics. Anatomically, stomatal density and the complexity of the area were also significantly affected by the choice of interstock. Overall, these findings highlight the important role of interstocks in modifying plant vigour, physiology, and nutrient acquisition. Future studies are needed to assess the long-term field performance of these combinations under various agro-climatic conditions.

## Introduction

Mango (*Mangifera indica* L.) is a major commercial fruit crop cultivated extensively in tropical and subtropical regions worldwide (Bompard, 2009). It is believed to have originated in the Indo-Burma region of Southeast Asia, particularly in the foothills of the Himalayas (Mukherjee, 1951). Many research studies indicate that mango possesses a wide range of health benefits, including antidiabetic, antioxidant, antiviral, cardiotonic, hypotensive, and anti-inflammatory properties (Yahia et al., 2023; Zapata-Londoño et al., 2020; Xu et al., 2013; Shah et al., 2010). In India, it holds significant cultural importance and is often referred to as the “King of fruits,” “Pride of Hindustan,” and “Bathroom fruit.” India is the leading mango-producing country, occupying 2.39 million hectares of land with a total annual production of around 22.39 million tons, and an average productivity of ~9.36t/ha (Anonymous, 2023). Despite being the world’s largest producer of mangoes, national productivity is lower than that of some industrialized nations for a variety of reasons. Low planting densities, large tree sizes, and the use of non-standardized rootstocks with unknown pedigrees can indeed contribute to lower productivity in mango orchards (Reddy and Raj, 2015). Typically, mango grafting research has shown that rootstocks have an impact on several scion physiological factors (Rosa et al., 2003). The biggest issue with mango cultivation is the lack of standardized rootstocks. Grafting is the ideal method for commercial use because it maintains the genetic purity of the propagated variety. Polyembryonic rootstocks play a crucial role in the commercial production of grafted mango plants because they are capable of producing both zygotic seedling (arising from sexual reproduction) and multiple nucellar seedlings (arising from the maternal tissue of the seed). These nucellar plantlets are genetically identical to the mother plant and exhibit uniform growth characteristics. Their abundance and uniformity make them ideal rootstocks, as they provide a reliable and consistent foundation for grafting superior scion varieties (Ravishankar et al., 2004).

While recent scientific endeavours have increasingly delved into understanding the genetic and physiological basis of root traits to improve crop productivity, grafting remains a practical and effective technique employed by farmers and horticulturists worldwide (Warschefsky et al., 2016). Rootstocks play a crucial role in influencing gas exchange (Wang et al., 2017; Loreti et al., 2002), tree vigour (Vahdati et al., 2021; Haak et al., 2006), fruit set, yield, and fruit quality (Dayal et al., 2016; Sotiropoulos, 2006). By selecting appropriate rootstocks, growers can optimize these factors to enhance overall productivity. For instance, rootstocks can regulate tree size, promoting manageable tree heights and higher planting densities, which are essential in high-density orchards (Webster, 2004; Tworkoski and Miller, 2007). Additionally, scion-stock interactions influence the exchange of gases such as carbon dioxide and water vapor, which directly impact photosynthesis, transpiration, and overall tree health (Hartmann et al., 2011). One of the most notable effects observed is the reduction of scion growth, which has been extensively studied withdwarfing rootstocks and interstocks (Gonçalves et al., 2003). As the leaf is a crucial organ for photosynthesis, its size and stomatal density play a vital role in determining photosynthetic efficiency (Schechter et al., 1991). These factors, in turn, influence the overall growth potential of the plant by affecting nutrient supply, resistance to environmental stresses, and the distribution of carbon to various plant organs.

Interstock or double graft has also been used with promising results to reduce plant height. This technique may also minimize incompatibility problems by using as intermediate stocks a cultivar compatible with the other two incompatible cultivar combinations (Ismail and Ebeed, 2013). Studies have shown that grafting onto dwarfing rootstocks or interstocks can effectively restrict plant height, crown size, and tree volume (Valdivia et al., 2000). However, it’s noteworthy that plants grafted onto vigorous rootstocks often exhibit enhanced nutritional properties but lower yields (Karlidag et al., 2014). This suggests a trade-off between growth vigour and fruit production, highlighting the importance of selecting the most appropriate graft combination based on specific environmental conditions and desired fruit quality (Valdivia et al., 2000). These interactions also affect fruit set and yield, ensuring better fruit retention and development (Bosa et al., 2014). Rootstocks can also enhance a plant’s resistance to factors like drought, salinity, and soil-borne pathogens, which are crucial for sustainable crop production in challenging growing conditions (Haroldsen et al., 2012; Davis et al., 2008). In other crops, such as apples (*Malus domestica*), the choice of rootstock significantly impacts scion growth habits, influencing the balance between reproductive and vegetative growth (Costes et al., 2003; Costes and García-Villanueva, 2007).

Leaf nutrient analysis provides a critical insight into the actual nutrient uptake and current nutritional status of fruit trees, serving as a valuable tool for growers to make informed decisions about orchard fertilizer requirements. Addressing the mineral nutrition of trees is essential, as fertilization is a pivotal factor in fruit production. Deficiency or toxicity of mineral elements can significantly hinder plant growth and development, leading to reduced yields and compromised fruit quality (White and Brown, 2010). Rootstock selection plays a vital role in nutrient uptake and utilization in grafted plants, directly impacting plant health, growth, and fruit quality (Bavaresco et al., 2003; Habran et al., 2016). Rootstocks influence the absorption, transport, and efficiency of mineral elements from the soil to the scion (Nawaz et al., 2016). These effects may be attributed to variations in root architecture, alterations in ion transporter activity, hormonal regulation, and miRNA expression (Meister et al., 2014; Zeng et al., 2014). Research has shown that different rootstocks can modify mineral uptake, transport, and assimilation. For instance, in apple trees, rootstocks like M9 and MM106 exhibit distinct capacities for absorbing nitrogen (N), manganese (Mn), iron (Fe), potassium (K), calcium (Ca), and phosphorus (P) (Amiri et al., 2014; Fallahi et al., 2001; Cheng and Raba, 2009). Similarly, grapevine rootstock architecture plays a key role in nitrogen use efficiency (NUE) and phosphorus (P) uptake (Zamboni et al., 2016). Additionally, Prunus rootstocks significantly influence macro- and micronutrient composition in cherry (Hrotko et al., 2014), peach (Mestre et al., 2015), and plum (Reig et al., 2018) leaves, as well as nutrient levels in cherry (Jimenez et al., 2004) and peach (Zarrouk et al., 2005) flowers. Studies have also shown that rootstocks significantly affect mineral uptake in pear (Pyrus communis L.) (North and Cook, 2006) and citrus (Zekri and Parsons, 1992). Monitoring the physiological and nutritional status of plants through leaf nutrient analysis is an established method (Baxter et al., 2008; Uçgun and Gezgin, 2017). This technique also facilitates the evaluation of interactions between scion and rootstock in terms of growth, nutrient uptake, and fruit yield (Ahmed et al., 2007; Jiménez et al., 2007; Savvas et al., 2011).

The rootstock effect on the performance of scion cultivars has been studied and demonstrated in many of the fruit crops, including mango, however no such studies related to the effect of interstock on grafting success, morpho-physio-chemical, and anatomical traits of mango have been conducted yet. Thus, the present study was conducted to evaluate the effect of different grafting combinations on morpho-physio-chemical parameters, nutrient contents of leaf and soil, and anatomical traits in mango.

## Materials and methods

### Plant materials

**Figure 1:**
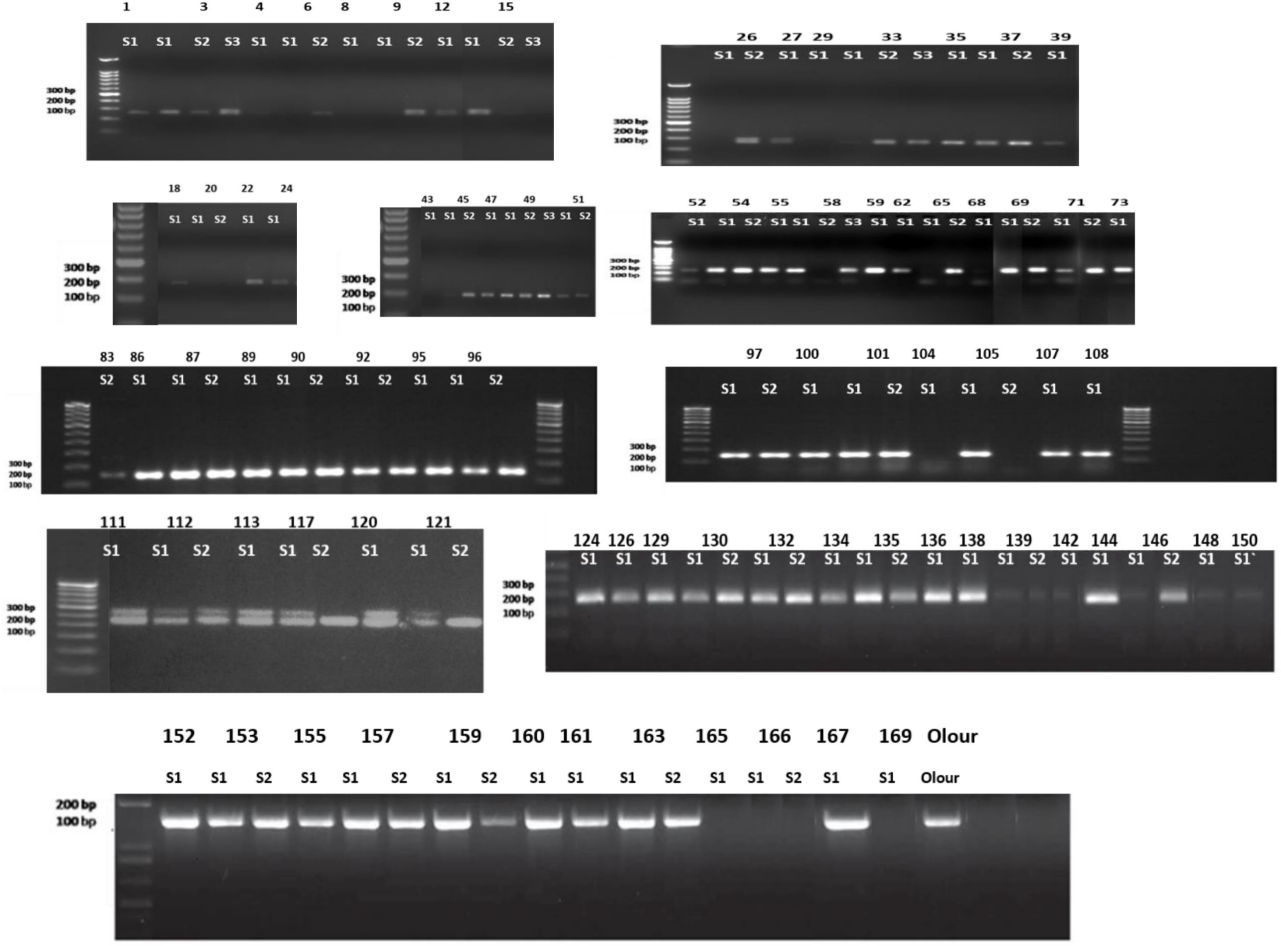
Screening of nucellar seedlings of Olour using SSR marker LMMA8.

**Figure 2:**
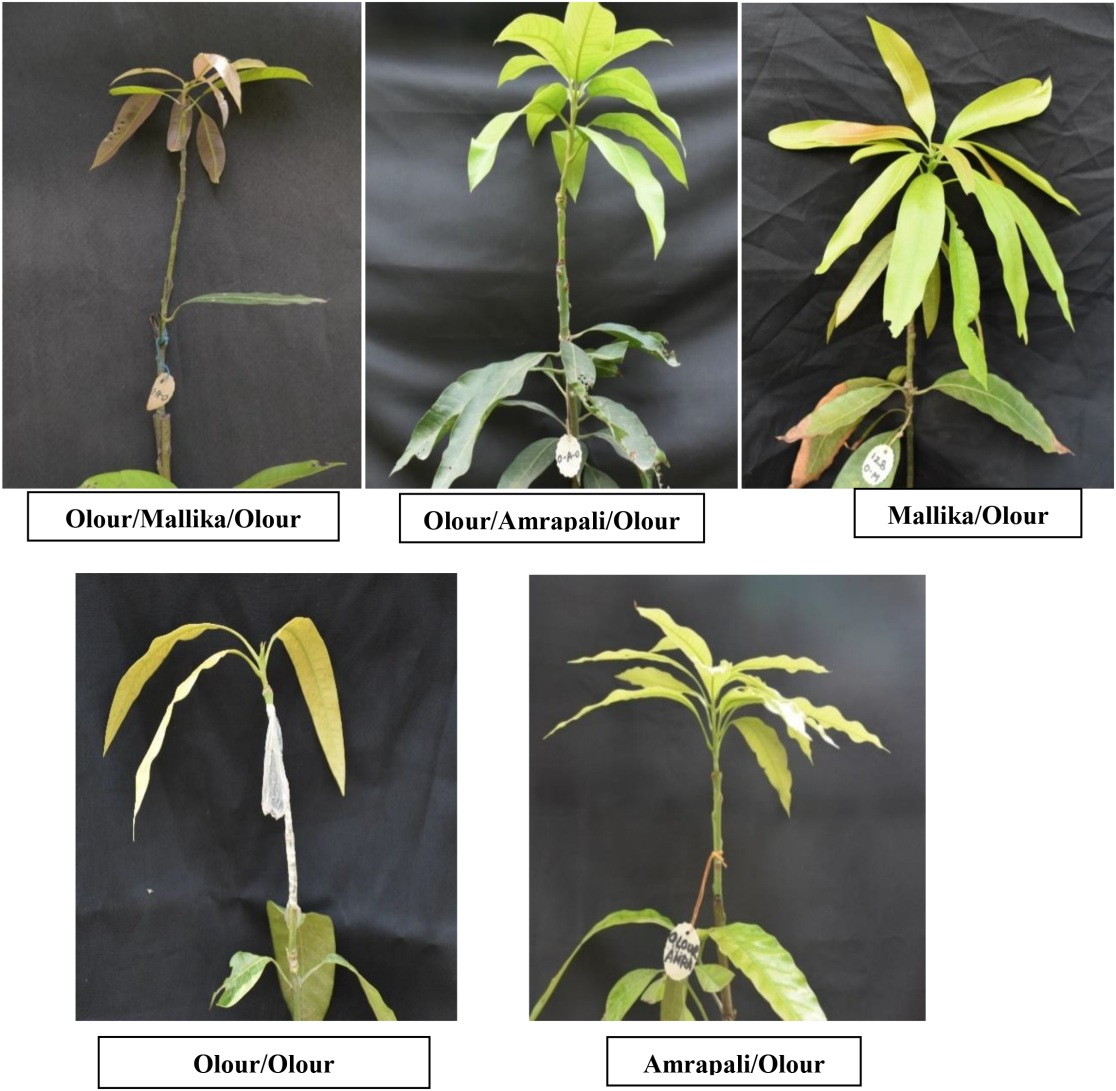
Different graft combinations after screening Olour seedling with SSR markers.

The present investigation was carried out in the Experimental Orchard of the Division of Fruits and Horticultural Technology, ICAR- IARI New Delhi, which is located at 77°07’E longitude, 28°41’N latitude, and an altitude of 227.4 m above mean sea level during the year 2021-24.The treatment consisted of various grafting combinations involving two nterstocks: no interstock, Amrapali (Dashehari x Neelum) as an interstock, and Mallika as an interstock (Neelum x Dashehari), with Olour used as both the rootstock and scion. The grafting combinations used in the experiment included both double and single grafts, as outlined in **Table 1**. In mango rootstock research in India, a major limitation is the absence of standardized clonal rootstocks and the lack of reliable methods for identifying true-to-type nucellar seedlings for grafting. This has resulted in inconsistency and non-uniformity across studies, leading to variable performance. Additionally, there is a notable gap in understanding the physiological and anatomical changes occurring at the graft union, especially when interstocks are used - an area that remains largely unexplored in mango. In polyembryonic mango rootstocks, one sexual embryo and multiple nucellar embryos are typically present, with the nucellar embryos possessing the same genetic makeup as the mother plant (Cordeiro et al., 2006). These adventitious embryos arise directly from the maternal nucellar tissue surrounding the embryo sac, which contains the developing zygotic embryo. Therefore, identifying the zygotic embryo is of critical importance in mango. LMMA8 primer was used in this study to detect polymorphism and to distinguish between nucellar and zygotic seedlings derived from stones of Olour rootstock. The forward primer sequence of LMMA8 marker was 5’-CATGGAGTTGTGATACCTAC-3’, and the reverse primer sequence was 5’-CAGAGTTAGCCATATAGAGTG-3’. The primer was designed to amplify at an annealing temperature of 47 °C. The identical SSR profiles of seedlings and their mother plants (Olour) confirm their nucellar origin, while any deviation from the mother plant’s SSR profile indicates a zygotic origin (**Figure 1**). Identified nucellar seedlings like seedling no. 152,155,167 etc. were subsequently grafted by interstocks and scions whereas zygotic seedlings like seedling no. 117, 112 etc. were discarded after screening from LMMA8 marker. The selection of Olour as a rootstock for this study is based on its well-documented salt tolerance, dwarfing ability, and genetic uniformity, making it a valuable choice for improving commercial mango cultivars in diverse environmental conditions (Dubey et al., 2007). Additionally, Olour exhibits good compatibility with most improved mango cultivars, including Langra, Himsagar, and Alphonso, providing greater flexibility in grafting combinations. One of Olour’s most significant traits is its polyembryonic nature, which ensures the production of nucellar seedlings that are genetically uniform and true-to-type. This study addresses this gap by selecting two contrasting mango hybrid cultivars–Amrapali (Dashehari x Neelum), known for its dwarfing habit, and Mallika (Neelum x Dashehari), recognized for its vigorous growth - as both interstocks and scions on a common rootstock (Olour). Amrapaliis precocious, distinctly dwarf, highly regular and prolific in bearing. It is suitable for high-density orcharding (Singh et al., 1972; Majumder et al., 1982). Mallika tree is semi-vigorous. It is medium to heavy cropper, and has a strong tendency to bear regularly. The fruits have an attractive appearance and the average fruit weight is 307 g (Singh et al., 1972). Both these hybrids original mother plants are present in ICAR- IARI experimental field (Both the hybrids were developed at ICAR-IARI). By using Amrapali and Mallika which are present on their own root system as well as in combination with Olour rootstock, the study aims to investigate how these genotypes influence scion vigour or dwarfness and to evaluate the graft-transmissible effects across the graft union. Initially ripened Olour fruits were harvested and then the stones were extracted. The extracted stones were sown in July 2022, with germination occurring within 30–40 days. After 3–4 months of seedling growth, molecular screening using SSR markers was conducted to identify true-to-type nucellar seedlings and eliminate zygotic ones. The first grafting was carried out in May 2023, followed by a second grafting in September 2023. Physiological, anatomical, and biochemical observations were recorded in 2024, ensuring sufficient growth period post-grafting- (**Figure 2**). Softwood grafting was performed in this study, and the plants were cultivated in the open field. The grafting was carried out on newly emerged flushes. Scion wood, having the same thickness as the terminal shoot, was defoliated 10 days prior to grafting. The grafts were securely tied using a 1.5 cm wide, 200-gauge polyethylene strip. In this technique, the graft union was positioned higher above the ground, and the rootstock sprouts were regularly pinched off as they have the potential to interfere with scion growth to ensure better quality. This foundational step validates the uniformity and reliability of the experimental material. For the double-grafted plants, the length of the interstock was uniformly maintained at 10 cm to ensure consistency across treatments. All cultural practices, including irrigation, weeding, hoeing, and fertilization, were performed uniformly across the treatments to ensure consistency and eliminate variability due to management practices.

**Table 1:** Grafting combinations used in the experiment.

S. no.	Grafting combination	Abbreviation	Rootstock	Interstock	Scion
1	Olour / Amrapali / Olour	O/A/O	Olour	Amrapali	Olour
2	Olour / Mallika / Olour	O/M/O	Olour	Mallika	Olour
3	Mallika / Olour	M/O	Olour	–	Mallika
4	Amrapali / Olour	A/O	Olour	–	Amrapali
5	Olour / Olour	O/O	Olour	–	Olour

### Soil nutrient status of the experimental site

The physicochemical analysis of soil from the experimental site revealed a nutrient-rich profile (**Table 2**). Macronutrient levels were high, with nitrogen at 2.78%, phosphorus 1.004%, calcium 3.85%, potassium 1.75%, and magnesium 0.45%. Micronutrients included zinc (369.74 ppm), copper (300.98 ppm), iron (15,878.14 ppm), manganese (381.13 ppm), boron (340.17 ppm), and sodium (19,889.11 ppm). Trace elements such as cobalt (4.97 ppm), aluminium (5938.86 ppm), chromium (32.57 ppm), nickel (16.68 ppm), cadmium (3.04 ppm), strontium (212.29 ppm), and lead (16.48 ppm) were also present. These values indicate that the soil was nutritionally sufficient to support healthy plant growth and allowed for a reliable assessment of grafting effects without confounding limitations from nutrient deficiencies.

**Table 2:** Soil nutrient status of experimental site of division of fruits and horticultural technology.

Nitrogen (%)	Phosphorus (%)	Calcium (%)	Potassium (%)	Magnesium (%)	Zinc (ppm)	Copper (ppm)	Iron (ppm)	Manganese (ppm)	Boron (ppm)	Sodium (ppm)	Cobalt (ppm)	Aluminium (ppm)	Chromium (ppm)	Nickel (ppm)	Cadmium (ppm)	Strontium (ppm)	Lead (ppm)
2.78	1.004	3.85	1.75	0.45	369.74	300.98	15878.14	381.13	340.17	19889.11	4.97	5938.86	32.57	16.68	3.04	212.29	16.48

### Physical parameters

The physical parameters of seedlings and leaves, viz., internodal length, leaf length, leaf width, leaf ratio, leaf fresh and dry weight, leaf area, no. of leaves, rootstock and scion girth, and stock: scion ratio were measured following the standard procedures. Girth of the rootstock was measured at 5 cm below the first graft union, while girth of the interstock and scion were measured at 5 cm above the first and second graft union, respectively with the help of vernier caliper. The stock-scion ratio was calculated by dividing the rootstock girth by the scion girth. To measure the average internodal length, the first 5 internodes from 60 cm height above the base of each individual plant were selected. The length of each internode was measured in cm using a measuring tape. Then, the lengths of all 5 internodes were added together to get the total length. Finally, the total length was divided by 5 to calculate the average internodal length for each plant, which was expressed in cm. Five fully mature leaves were collected from grafted plants, and their length and width were measured using a vernier caliper, with the results expressed in cm. The leaf ratio was determined using the measured values of leaf length and width. Leaf area was measured with WINFOLIAPro2020, aleaf area meter, and expressed in cm^2^. Leaf fresh weight and dry weight (leaves were dried at 65˚C in hot air oven for 72 hours until the weight becomes constant)were measured using an electronic balance. Number of leaves was counted manually from each grafting combination.

### Foliar photosynthetic pigments

Chlorophyll (‘a’, ‘b’, total) and carotenoids were estimated using the method described by Hiscox and Israelstam (1979). A fully opened mature leaf was taken as the experimental sample for chlorophyll estimation. Leaf samples weighing 50 mg were collected, then cut into small pieces and placed in a test tube. 10 ml of dimethyl sulphoxide (DMSO) was added to it, and the solution was kept in an oven at 60-62˚C for 4 hours. The following day, readings were taken at 480 nm, 649 nm, and 665 nm, respectively, through UV UV-Visible spectrophotometer (UV PLUS, MOTRAS SCIENTIFIC). Chlorophyll ‘a’, ‘b’, and total carotenoid content were estimated according to the formula given by Wellburn, 1994. The formulas are given below:


Chlorophyll ‘a'=(12.19 ×A 665)−(3.45×A 649) μg/mlChlorophyll ‘b'=(21.99 ×A 649)−(5.32×a 665) μg/mlTotal chlorophyll=Chl'a'+Chl'b'Total carotenoids = [1000× A 480−(2.14 × Chl a)−(70.16× Chl b)220 μg/ml

### Leaf gas exchange parameters and membrane stability index (%)

Gas exchange measurements were conducted on fully opened mature leaves from each genotype, with a minimum of five observations recorded between 09:00 and 11:30 h. The observations were made under ambient light and CO_2_ levels. Mature leaves were placed inside an infrared gas analyzer (IRGA) (LICOR, Lincoln, NE, USA) until the relative humidity (RH) and internal leaf CO_2_ concentration (Ci) reached stable values. Net photosynthetic rate (*A*) (μmole CO_2_ m^-2^ s^-1^), transpiration rate (*E*) (m.mole H_2_O m^-2^), stomatal conductance (*gs*) (mole H_2_O m^-2^s^-1^), and internal leaf CO_2_ concentration (*Ci*) (μmole CO_2_ m^-2^ s^-1^) were measured. Data were transferred to a computer for analysis. Observations on gas exchanges were recorded under a photoperiod of 11 hr, optimum day temperature of 32.7°C & night temperature, 18.9°C, and relative humidity of 71.9%. Using the Sairam et al. (1994) approach, the membrane stability index (MSI) was computed by measuring the electrical conductivity of leaf leachates in double-distilled water at two different temperatures: 40°C and 100°C. Small pieces of mature leaf, weighing 0.5 g, were chopped up and placed in two sets of test tubes containing 10 ml of double-distilled water. The electrical conductivity of both sets, denoted as C1 and C2, was measured using anelectric conductivity meter. One set of test tubes was maintained at 40°C for 30 minutes, while the other set was heated to 100 °C in a boiling water bath for 15 minutes. The electrical conductivity measurements before and after heating provide insights into the membrane stability of the leaves. The membrane stability index (MSI) was then calculated using the formula:


MSI (%)=1−[(C1/C2)×100]

### Biochemical parameters

#### Total sugar content in leaves

The total sugar content of the leaf tissue was determined using the Anthrone reagent method outlined by Sadasivam and Manickam in 1992 (Sadasivam and Manickam, 1992). In this method, freshly cut leaf samples weighing 0.2 g were homogenized in 80% ethanol (v/v) using a boiling water bath for 1 hour. The supernatant obtained after filtration through filter paper (Whatman No. 1) was collected, and this process was repeated twice. The collected supernatant was then boiled with double-distilled water (ddw) to adjust the volume to 50 ml. To ascertain the sugar content, 4 ml of freshly prepared Anthrone reagent was added to 1 ml of the sugar sample. The mixture was heated over a boiling water bath for 8 minutes and then allowed to cool. The optical density of the resulting green to dark green colour solution was measured at 630 nm, indicating concentration. The concentration of total sugars was calculated by plotting the optical density (OD) values on a graph constructed using glucose as a standard. The results were expressed as mg g^-1^ of the sample.

#### Total soluble protein content in leaves

The total soluble protein content was determined using Bradford’s protein assay technique, as described by Bradford in 1976 (Bradford, 1976). This method relies on the principle that Coomassie Brilliant Blue G250 undergoes an absorbance shift from 465 to 595 nm in an acidic environment upon binding with proteins. To create a standard curve, a Bovine Serum Albumin (BSA) stock solution of concentration 100 μg/ml was prepared, and dilutions ranging from 0 to 100 μg/ml were made. For the protein estimation, a reaction mixture was prepared by combining 2 ml of Bradford reagent, 990 ml of water, and 10 ml of enzyme extract. The reaction mixture was allowed to incubate in the dark for 10 minutes. Following the incubation period, the absorbance of the reaction mixture was measured at 595 nm using a SHIMADZU UV-1900i spectrophotometer. The concentration of protein was then determined by comparing the absorbance values to the standard curve, and the protein content was expressed in mg/ml.

#### Leaf proline content

To determine the proline content in the leaf sample, a procedure based on the method outlined by Bates et al. (1973) was followed. Initially, 0.5 g of the leaf sample was homogenized in 5 ml of sulphosalicylic acid (3%) using a mortar and pestle. The resulting homogenate was then filtered through Whatman No. 1 filter paper to obtain the filtrate, which served as the basis for proline content determination. To estimate proline, 2 ml of the filtrate was mixed in a test tube with 2 ml of glacial acetic acid and 2 ml of Ninhydrin reagent. The reaction mixture was then heated in a boiling hot water bath at 100 °C for 30 minutes. Subsequently, a brick-red colour developed suddenly upon heating. After allowing the mixture to cool, 6 ml of toluene was added, and the solution was transferred to a separating funnel. The mixture was thoroughly mixed, and the chromophore-containing toluene layer was separated. The absorbance of the toluene layer was measured at 520 nm using a SHIMADZU UV-1900i spectrophotometer against a toluene blank. The concentration of proline in the sample was estimated by referring to a standard curve constructed from known concentrations of proline.


$$\text{Proline}\;(\mu \text{g}\;{\text{g}}^{-1}\text{of}\;\text{FW})=\frac{\text{Proline conc. (μg/ml)}\times \text{volume of toluene (ml)}\times 5\;}{115.5\times \text{weight}\;\text{of}\;\text{the}\;\text{sample}\;(\text{g})}$$

#### Apical bud phenols

In the Folin-Ciocalteu method (Singleton et al., 1999) for determining total phenol content, a 2g leaf sample was accurately weighed and ground with 20 ml of 80% ethanol in a pre-chilled pestle and mortar. After grinding, the sample was centrifuged for 20 minutes at 10,000 rpm to separate the supernatant, which was then collected for further analysis. In a test tube, a 100µl aliquot of the supernatant was combined with 2.9 ml of distilled water and 0.5 ml of Folin-Ciocalteu reagent. After allowing the mixture to sit for three minutes, 2 ml of sodium carbonate (Na_2_CO_3_) solution was added and thoroughly mixed. The absorbance readings were then taken using a UV-VIS double-beam PC 8 scanning auto cell spectrophotometer at a wavelength of 765 nm.

### Leaf anatomy

#### Stomatal density and other stomatal parameters

On a bright day, five completely developed leaves were collected from each combination. The leaf samples were cleansed with double-distilled water to remove any impurities. Subsequently, samples of leaves with three to four veins along the borders were treated with transparent glue and allowed to cure. Once dried, the leaf membrane was carefully removed and placed on a microscope slide for 15 to 20 minutes before being covered with a coverslip. Using a simple microscope (such as OLYMPUS CX33) and imaging software (Mag vision software), the abaxial surface of the leaf was observed keenly (Sampson, 1961). Slides were examined under a low-power objective to identify the densest patches of stomata. Stomatal density was determined by counting the number of stomata per field, typically 0.25 mm^2^, under 10X magnification and 50X magnification.In leaf parameters pore length (µm), pore width (µm), pore area (µm^2^), right and left arc length (µm), stomatal complex length and width (µm), stomatal complex area (µm^2^), potential stomatal conductance index and perimeter of stomata (µm) were measured.Pore area and potential stomatal conductance index was calculated by using the following formulaes:


$$\begin{array}{l} \text{Pore area}=\text{pore length}\times \text{pore width}\\ \text{Potential stomatal conductance index = pore area }\times \;\text{stomata density }\times 0.0001 \end{array}$$

#### Macronutrients, micronutrients, and heavy metals estimation in soil and leaf

Soil samples were typically collected from the root zone at multiple locations (6 locations) within the field to account for spatial variability. The collected samples were thoroughly mixed to form a composite sample, which was air-dried in the shade to preserve nutrient integrity. Then the soil samples were dried in hot air oven at 65°C for 72 hours until a constant weight was achieved. Once dried, the soil was sieved through a 2 mm mesh to remove debris and prepared for laboratory analysis. For macronutrient and micronutrient analysis, soil samples were subjected to various chemical extraction methods. Nitrogen is typically measured using the Kjeldahl method, while phosphorus was analyzed using the Olsen or Bray extraction method, depending on soil pH (Olsen et al., 1954). Hard green leaves from the latest flush of all grafting combinations were collected for nutrient analysis. The samples were thoroughly washed with tap water, followed by deionized water, then blot-dried and oven-dried at 65 °C until a constant weight was achieved (approximately 48 hours). After drying, the samples were ground and sent to a soil laboratory for analysis. Total nitrogen (N) was determined using the Dumas high-temperature combustion method with a Leco analyzer. To estimate phosphorus (P), potassium (K), sodium (Na), calcium (Ca), magnesium (Mg), micronutrients (B, Fe, Mn, Cu, Zn), and heavy metals (Co, Al, Cr, Ni, Cd, Sr, Pb), 1 g of the plant sample was digested in a 4:1 mixture of nitric acid and perchloric acid (HNO_3_: HClO_4_). Potassium (%) and sodium (%) were analyzed using a flame photometer (Systronics Flame Photometer 128) (Jackson, 1973). Other elements, including Ca, Mg, B, Cu, Fe, Mn, Co, Al, Cr, Sr, Cd, Pb, Ni, and Zn, were quantified using inductively coupled plasma mass spectrometry (ICP-MS) following acid digestion (USEPA, 2014).

### Statistical analysis

The experiment was conducted using a Randomized Block Design (RBD) with five treatments and five replications (n=5), with each replication containing a single seedling. The data were subjected to statistical analysis of variance (ANOVA) using R software, and significant differences were compared, followed by DMRT at p ≤ 0.05. The analysis of data was used to interpret the results and draw valid conclusions. Correlation of coefficients, clustering, heat maps, and PCA among the physical parameters, stomatal parameters, leaf nutrient contents, photosynthetic pigments, physiological and biochemical parameters were calculated and prepared using the R software.

## Results

A comprehensive evaluation was conducted to assess the impact of different grafting combinations on a range of traits. These included physical parameters, stomatal characteristics influencing gas exchange, and foliar photosynthetic pigments (chlorophyll a, chlorophyll b, total chlorophyll, and carotenoids), which are critical indicators of photosynthetic efficiency. Detailed anatomical observations were also made on leaf and stomatal structures to understand structural adaptations. In addition, macro- and micronutrient contents were quantified to assess nutrient uptake efficiency, while physiological parameters such as photosynthesis rate, transpiration, stomatal conductance, and membrane stability index were analyzed to evaluate plant metabolic performance. Biochemical traits were also included to provide insight into the plant’s functional responses. Together, these parameters were systematically analyzed and compared among the grafting combinations to identify combinations that promote optimal vegetative growth and physiological functioning.

### Physical parameters

Analysis of the data presented in **Table 3** reveals that rootstock girth, scion girth and internodal length were significantly affected by the different grafting combinations. However, no significant differences were observed in the stock-to-scion ratio across the various grafting combinations. Rootstock girth ranged from 7.11 mm in the Olour/Amrapali/Olour combination to 10.28 mm in the Mallika/Olour combination, with an overall mean of 8.23 mm. The Mallika/Olour combination also recorded the highest scion girth (6.33 mm), whereas the Amrapali/Olour/Amrapali combination exhibited the lowest (4.32 mm), with a mean value of 5.01 mm across different grafting combinations. The stock-to-scion ratio remained relatively stable, ranging from 1.63 in Mallika/Olour to 1.65 in other grafting combinations, with a mean of 1.64. Internodal length varied notably among combinations, with the Mallika/Olour grafts showing the greatest length (1.67 cm), indicating enhanced vegetative vigour, while the Amrapali/Olour grafts recorded the shortest internodes (0.93 cm), reflecting a tendency toward dwarfing.

**Table 3:** Effect of rootstock and interstock on rootstock girth, scion girth, stock:scion ratio and internodal length.

Grafting combination	Rootstock girth (mm)	Scion girth (mm)	Stock: Scion girth	Internodal length (cm)
M/O	10.28^a^	6.33^a^	1.63^a^	1.67^a^
O/O	8.05^b^	4.90^b^	1.65^a^	1.15^c^
A/O	7.32^c^	4.42^c^	1.65^a^	0.93^d^
O/A/O	7.11^c^	4.32^c^	1.65^a^	1.30^bc^
O/M/O	8.37^b^	5.08^b^	1.65^a^	1.33^b^
Mean	8.23	5.01	1.64	1.28
CV	5.90	6.23	6.57	6.97
SE (m) ±	0.22	0.14	0.05	0.04
LSD (p ≤ 0.05)	0.65	0.410	NS	0.12

Superscript in small letters on the value of each parameter indicates significant difference at *p ≤ 0.05.*

**Table 4** further highlights significant differences in leaf morphology and biomass attributes among the grafting combinations, such as leaf length, width, ratio, fresh and dry weight, area, and number of leaves. The longest leaves (17.85 cm) were observed in the Mallika/Olour combination, while the shortest (9.67 cm) were recorded in Olour/Olour, with an average length of 12.45 cm. Leaf width ranged from 2.28 cm in Olour/Olour to 3.71 cm in Olour/Mallika/Olour, with a mean of 3.18 cm. The highest leaf length-to-width ratio (5.17) was foundin Mallika/Olour, and the lowest (3.38) in Olour/Mallika/Olour. The Mallika/Olour treatment also exhibited the greatest leaf fresh weight (3.03 g) and dry weight (1.11 g), whereas Olour/Amrapali/Olour had the lowest values (1.26 g and 0.40 g, respectively). In terms of leaf area and number of leaves, the Mallika/Olour combination again outperformed the others with values of 56.48 cm² and 20.20 leaves, while Olour/Mallika/Olour recorded the lowest no. of leaves i.e.6.20 leaves and minimum leaf area was reported in Amrapali/Olour (26.83 cm^2^). These findings underscore the substantial impact of grafting combinations on vegetative growth and physiological performance in mango.

**Table 4:** Effect of rootstock and interstock on leaf parameters.

Grafting combination	Leaf length (cm)	Leaf width (cm)	Leaf ratio	Leaf fresh weight (g)	Leaf dry weight (g)	Leaf area (cm^2^)	No. of leaves
M/O	17.85^a^	3.54^ab^	5.17^a^	3.03^a^	1.11^a^	56.48^a^	20.20^a^
O/O	9.67 ^e^	2.28^d^	4.24^b^	2.15^b^	0.81^b^	34.30^b^	12.20^b^
A/O	10.09^d^	2.95^c^	3.41^d^	1.72^c^	0.41^d^	26.83^d^	11.00^b^
O/A/O	12.07^c^	3.42^b^	3.52^cd^	1.26^d^	0.40^d^	36.93^b^	15.40^ab^
O/M/O	12.59 ^b^	3.71^a^	3.38^d^	2.68^a^	0.51^c^	45.19^ab^	6.20^c^
Mean	12.45	3.18	3.94	2.17	0.65	39.95	13.00
CV	2.15	10.93	8.53	2.19	3.46	4.60	12.69
SE (m) ±	0.12	0.15	0.15	0.2	0.01	0.82	0.73
LSD (p ≤ 0.05)	0.35	0.46	0.45	0.6	0.03	2.46	2.21

Superscript in small letters on the value of each parameter indicates significant difference at *p ≤ 0.05*.

### Physiological traits

The data presented in **Table 5** demonstrates significant differences in physiological parametersnamely, intercellular CO_2_ concentration, stomatal conductance, net photosynthesis, transpiration, and leaf membrane stability index (MSI)among the various mango grafting combinations. The Olour/Mallika/Olour combination exhibited the highest intercellular CO_2_ concentration (356.20 µmol m^-2^ s^-1^), nearly 1.7 times greater than the lowest value observed in Olour/Amrapali/Olour (209.00 µmol m^-2^ s^-1^). The overall average across grafting combinations was 260.00 µmol m^-2^ s^-1^. A similar trend was evident in stomatal conductance, with Mallika/Olour showing the highest rate (0.070mol m^-2^ s^-1^), compared to just 0.014 mol m^-2^ s^-1^ in Olour/Amrapali/Olour. The net photosynthetic rate followed a similar pattern, with Olour/Mallika/Olour recording the highest value (8.51 µmol m^-2^ s^-1^) and Olour/Amrapali/Olour the lowest (3.50 µmol m^-2^ s^-1^). Transpiration rates varied from 0.36 mmol m^-2^ s^-1^ in Olour/Amrapali/Olour to 1.98 mmol m^-2^ s^-1^ in Olour/Mallika/Olour, with a mean of 1.00 mmol m^-2^ s^-1^. The leaf membrane stability index (MSI), an important indicator of cellular integrity and resilience, varied significantly among the grafting combinations. The Olour/Olour combination recorded the lowest MSI value (56.02%), whereas the Olour/Amrapali/Olour combination exhibited the highest (68.43%), indicating greater membrane stability and potential adaptability under suboptimal conditions. However, despite its higher MSI, the Olour/Amrapali/Olour combination showed comparatively lower photosynthetic efficiency, which may correlate with reduced vegetative vigour and a more compact growth habit. In contrast, the Olour/Mallika/Olour combination demonstrated superior performance in key physiological parameters, including higher rates of photosynthesis and transpiration, which are typically associated with enhanced plant vigour and biomass accumulation.

**Table 5:** Effect of rootstock and interstock on physiological parameters.

Grafting combination	Intercellular CO_2_ concentration (µmol m^-2^ s^-1^)	Stomatal conductance (mol m^-2^ s^-1^)	Leaf net photosynthesis (µmol m^-2^ s^-1^)	Transpiration (mmol m^-2^ s^-1^)	Leaf membrane stability index (MSI) (%)
O/O	239.40^c^	0.040^b^	5.34^c^	0.86^c^	56.02^d^
A/O	219.00^d^	0.023^c^	4.14^d^	0.54^d^	64.82^b^
M/O	276.40^b^	0.070^a^	6.02^b^	1.26^b^	60.25^c^
O/A/O	209.00^e^	0.014^b^	3.50^e^	0.36^e^	68.43^a^
O/M/O	356.20^a^	0.043^d^	8.51^a^	1.98^a^	62.25^bc^
Mean	260.00	0.038	5.50	1.00	62.48
CV	4.04	6.353	3.71	2.23	1.31
SE (m) ±	0.64	0.001	0.01	0.01	0.08
LSD (p ≤ 0.05)	1.94	0.003	0.03	0.04	0.26

Superscript in small letters on the value of each parameter indicates significant difference at *p ≤ 0.05*.

### Biochemical traits

Biochemical analysis of the leaves demonstrated significant variations among grafting combinations in terms of total soluble protein, sugars, phenols, and proline content (**Table 6**). The Olour/Mallika/Olour combination recorded the highest levels of total soluble protein (4.34 mg/g FW) and total sugars (119.05 mg/g FW), indicating greater metabolic activity and potentially vigorous growth. In contrast, the Olour/Amrapali/Olour combination showed markedly higher levels of phenols (3067.53 mg/100 g) and proline (1.06 μg/g FW) in the apical buds. These elevated levels are often associated with reduced cell division and elongation, and may play a regulatory role in growth inhibition. This biochemical profile aligns with the dwarfing trait observed in this grafting combination, suggesting that higher accumulation of phenolic compounds and proline could be contributing factors to its compact and restrained growth habit. The Olour/Mallika/Olour combination recorded the lowest proline (0.36 μg/g FW) levels and the lowest phenolic content (1014.31 mg/100 g). These findings underscore the influence of rootstock and interstock on the biochemical composition of mango leaves. Variations in nutrient uptake efficiency, and overall physiological performance likely contribute to the differences observed. The elevated protein and sugar levels in the Olour/Mallika/Olour combination suggest enhanced photosynthetic efficiency and nutrient assimilation, both of which are indicative of higher metabolic vigour and active growth. Such a biochemical profile typically supports robust vegetative development and overall plant vitality.Conversely, the Olour/Amrapali/Olour combination exhibited increased phenol and proline levels, which are known to influence growth regulation by modulating cellular processes such as lignification, osmotic adjustment, and cell expansion. The accumulation of these compounds may contribute to a more controlled or restrained growth pattern, resulting in reduced plant vigour. These findings emphasize the potential of specific grafting combinations for optimizing biochemical composition, which could be harnessed to improve plant performance under varying environmental conditions.

**Table 6:** Effect of rootstock and interstock on total soluble protein, sugars, phenols and proline content of leaves.

Grafting combination	Leaf total soluble protein (mg/g FW)	Leaf total sugars (mg/g FW)	Apical bud phenols (mg/100 g)	Leaf proline content (μg g^-1^ of FW)
O/O	2.24^c^	76.32^e^	1769.27^c^	0.53^d^
A/O	1.77^d^	94.73^c^	2816.23^b^	0.96^b^
M/O	2.81^b^	105.12^b^	1252.17^d^	0.73^c^
O/A/O	1.25^e^	81.84^d^	3067.53^a^	1.06^a^
O/M/O	4.34^a^	119.05^a^	1014.31^e^	0.36^e^
Mean	2.48	95.41	1983.9	0.73
CV	10.79	12.69	7.45	3.05
SE (m) ±	0.12	0.82	1.78	0.01
LSD (p ≤ 0.05)	0.36	2.44	5.34	0.02

Superscript in small letters on the value of each parameter indicates significant difference at *p ≤ 0.05*.

### Foliar photosynthetic pigments

**Figure 3:**
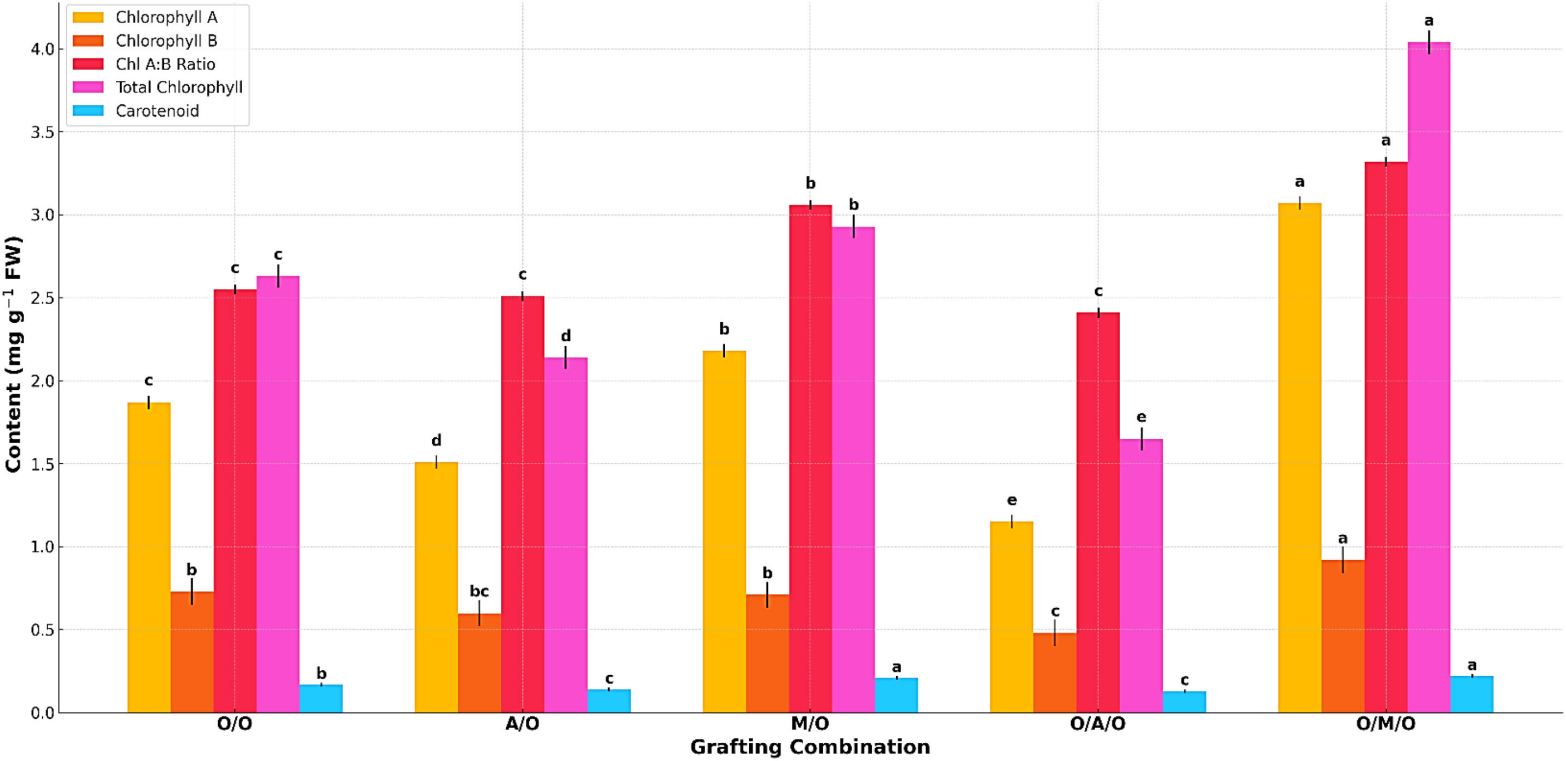
Effect of rootstock and interstock on foliar photosynthetic pigments.

As presented in **Table 7**, the levels of chlorophyll and carotenoids varied significantly among the grafting combinations, highlighting the influence of rootstock and interstock on foliar pigment composition. The Olour/Mallika/Olour combination consistently exhibited the highest concentrations of chlorophyll a (3.07 mg/g FW), total chlorophyll (4.04 mg/g FW), and carotenoids (0.22 mg/g FW), indicating enhanced photosynthetic efficiency and a greater capacity for light absorption. In contrast, the Olour/Amrapali/Olour combination showed the lowest levels of chlorophyll A (1.15 mg/g FW), total chlorophyll (1.65 mg/g FW), and carotenoids (0.13 mg/g FW), suggesting a relatively lower photosynthetic potential.Chlorophyll B content varied from 0.48 mg/g FW in Olour/Amrapali/Olour to 0.92 mg/g FW in Olour/Mallika/Olour, with the chlorophyll A:B ratio ranging between 2.41 and 3.32. These differences reflect variation in light-harvesting capacity and energy transfer efficiency among the grafting treatments. The superior pigment content observed in the Olour/Mallika/Olour combination suggests a physiological advantage that may contribute to improved growth and productivity. **Figure 3** complements the findings on foliar photosynthetic pigments, depicting that the Olour/Mallika/Olour combination has significantly higher levels of chlorophyll ‘A’, chlorophyll ‘B’, total chlorophyll, and carotenoids. These pigments are directly associated with photosynthetic performance, indicating that this graft combination is physiologically more efficient.

**Table 7:** Effect of rootstock and interstock on chlorophyll and carotenoid content of leaves.

Grafting combination	Chlorophyll A (mg g^−1^ FW)	Chlorophyll B (mg g^−1^FW)	Chlorophyll A:B	Total chlorophyll (mg g^−1^FW)	Total carotenoid content (mg g^−1^ FW)
O/O	1.87^c^	0.73^b^	2.55^c^	2.63^c^	0.17^b^
A/O	1.51^d^	0.60^bc^	2.51^c^	2.14^d^	0.14^c^
M/O	2.18^b^	0.71^b^	3.06^b^	2.93^b^	0.21^a^
O/A/O	1.15^e^	0.48^c^	2.41^c^	1.65^e^	0.13^c^
O/M/O	3.07^a^	0.92^a^	3.32^a^	4.04^a^	0.22^a^
Mean	1.96	0.69	2.77	2.67	0.17
CV	4.55	12.56	13.31	5.85	5.67
SE (m) ±	0.04	0.08	0.03	0.07	0.01
LSD (p ≤ 0.05)	0.12	0.24	0.09	0.21	0.02

Superscript in small letters on the value of each parameter indicates significant difference at *p ≤ 0.05.*

### Leaf anatomical parameters

**Figure 4:**
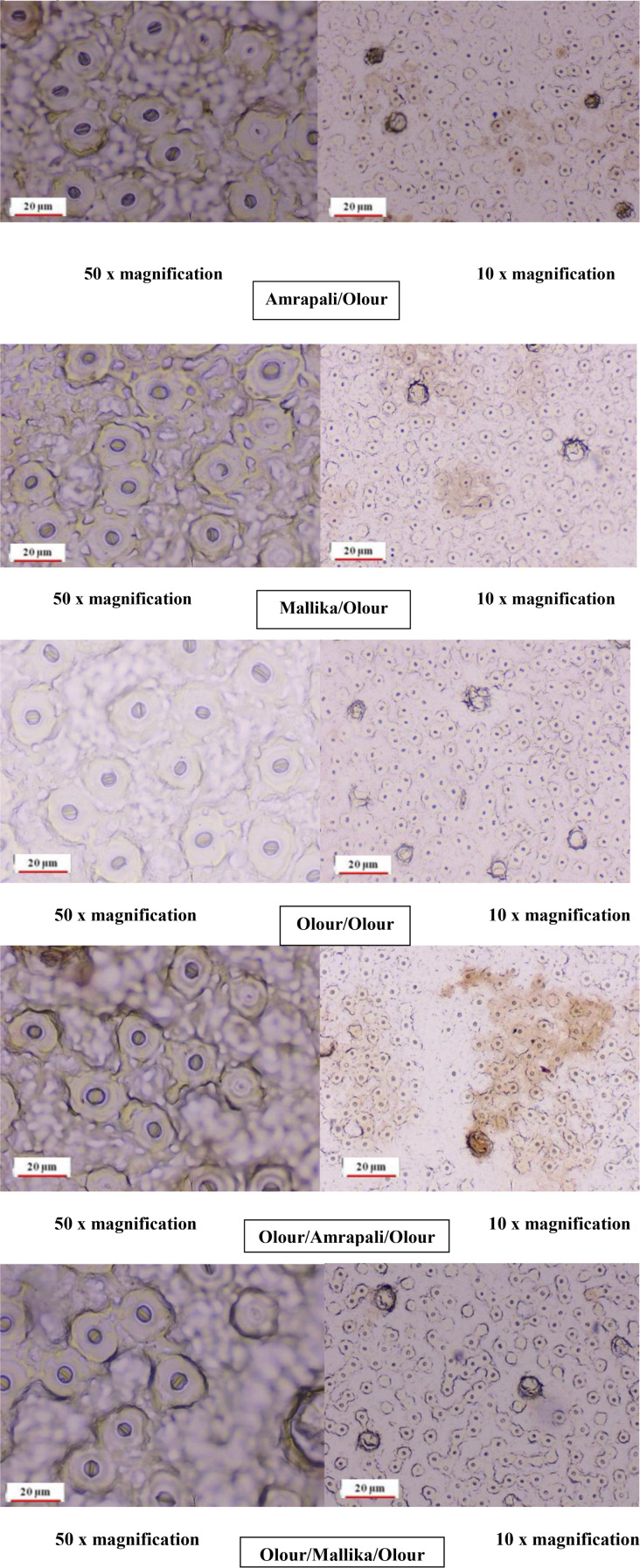
Stomatal distributions of the different grafting combinations at 10x and 50x magnification.

Anatomical analysis of stomatal traits (**Figure 4**; **Tables 8**, **9**) revealed significant differences among the grafting combinations, underscoring the influence of rootstock and interstock on stomatal structure and potential gas exchange efficiency. The Mallika/Olour combination exhibited the most prominent stomatal pore characteristics, with the longest pore length (78.31 µm), greatest pore width (72.90 µm), and the largest pore area (5708.57 µm²). In contrast, Olour/Amrapali/Olour recorded the smallest values for all three parameters - 65.60 µm length, 59.18 µm width, and 3883.55 µm² area - suggesting reduced gas diffusion potential. These variations reflect the influence of rootstock and interstock combinations on stomatal morphology, which may play a role in regulating gas exchange and water use efficiency. Regarding stomatal complex dimensions, the Mallika/Olour combination exhibited the largest complex length (260.32 µm), width (248.95 µm), and area (64806.98 µm²), suggesting greater potential for efficient gas exchange and photosynthesis. In contrast, the Olour/Olour combination showed the smallest dimensions (191.65 µm length, 179.76 µm width) and the smallest stomatal complex area (34454.50 µm²). The highest stomatal density was recorded in Olour/Mallika/Olour (746.00 mm^-2^), which also demonstrated the greatest potential stomatal conductance index (0.00031), indicating enhanced capacity for CO_2_ diffusion, transpiration and increased plant vigour. The perimeter of the stomata was found to be highest in Mallika/Olour (237.48 µm) and lowest in Amrapali/Olour (199.76 µm).

**Table 8:** Effect of rootstock and interstock on stomatal pore length, width, area, left and right arc length.

Grafting combination	Pore length (µm)	Pore width (µm)	Pore area (µm^2^)	Right arc length (µm)	Left arc length (µm)
O/O	74.28^b^	68.40^b^	5081.13^b^	303.53^d^	326.49^c^
A/O	70.50^c^	64.98^c^	4581.62^c^	360.80^bc^	355.35^ab^
M/O	78.31^a^	72.90^a^	5708.57^a^	376.86^ab^	380.94^a^
O/A/O	65.60^d^	59.18^e^	3883.55^e^	310.62^d^	344.67^bc^
O/M/O	67.96^d^	61.95^d^	4210.11^d^	392.73^a^	336.35^c^
Mean	71.33	65.48	4692.99	348.91	348.76
CV	6.51	7.57	14.05	10.44	5.47
SE (m) ±	0.28	0.35	0.15	0.14	0.34
LSD (p ≤ 0.05)	0.86	1.07	0.46	0.42	1.04

Superscript in small letters on the value of each parameter indicates significant difference at *p ≤ 0.05*.

**Table 9:** Effect of rootstock and interstock on stomatal complex length, width, area, density, potential stomatal conductance index and stomatal perimeter.

Grafting combination	Stomatal complex length (µm)	Stomatal complex width (µm)	Stomatal complex area (µm^2^)	Stomatal density (mm^−2^)	Potential stomatal conductance index	Perimeter of stomata (µm)
O/O	191.65^e^	179.76^e^	34454.50^e^	516.00^c^	0.00023^a^	224.11^b^
A/O	219.58^d^	202.85^d^	44544.72^d^	458.875^d^	0.00024^a^	199.76^e^
M/O	260.32^a^	248.95^a^	64806.98^a^	603.00^b^	0.00022^a^	237.48^a^
O/A/O	238.60^c^	219.14^c^	52288.06^c^	380.75^e^	0.00023^a^	226.58^ab^
O/M/O	250.99^b^	238.86^b^	59954.19^b^	746.00^a^	0.00031^a^	218.15^c^
Mean	232.23	217.91	51209.69	540.91	0.00024	216.95
CV	10.74	11.64	14.71	13.33	3.29	6.36
SE (m) ±	0.43	0.57	1.08	1.08	0.001	0.28
LSD (p ≤ 0.05)	1.31	1.72	3.25	3.25	NS	0.84

Superscript in small letters on the value of each parameter indicates significant difference at *p ≤ 0.05*.

These findings indicate that stomatal anatomical traits are significantly influenced by grafting combinations, with direct implications for photosynthetic activityand water-use efficiency. The larger pore size and complex dimensions observed in Mallika/Olour highlight its suitability for maximizing gas exchange, while the higher stomatal density recorded in Olour/Mallika/Olour underscores its potential for promoting physiological efficiency and contributing to increased plant vigour.

### Macronutrients, micronutrients, and heavy metals concentration

**Figure 5:**
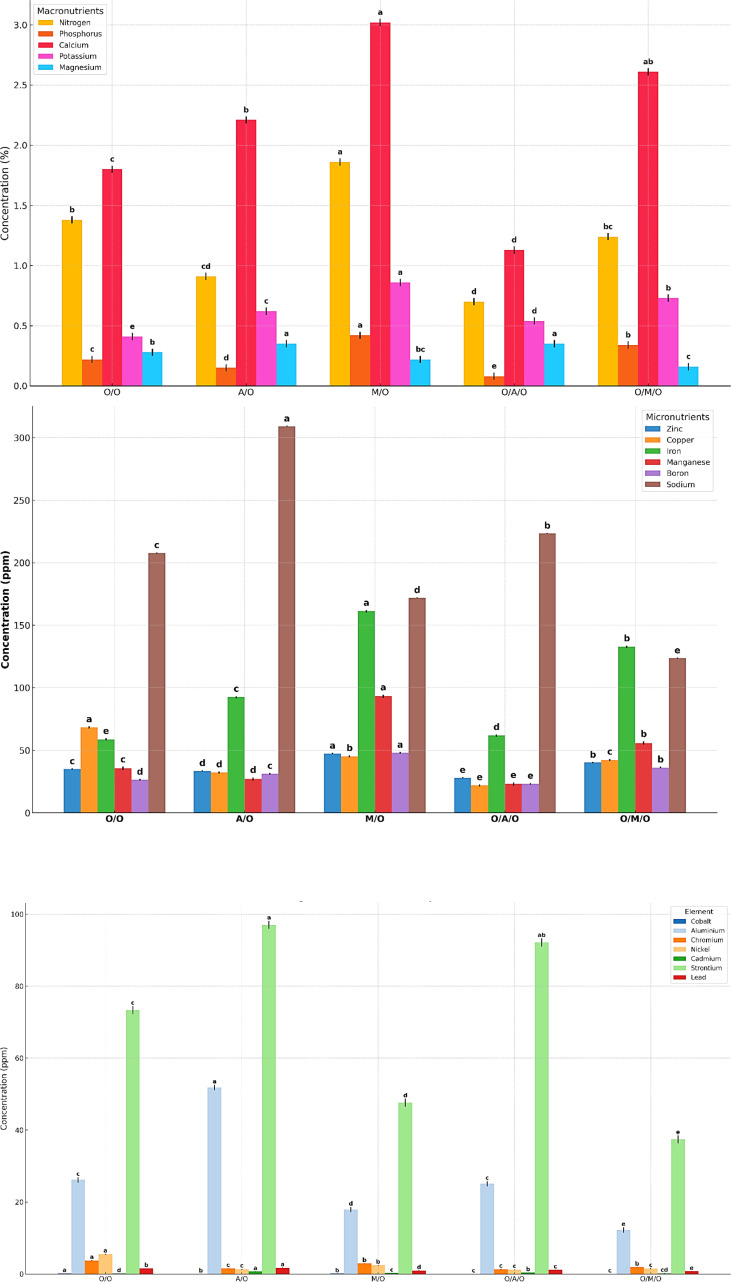
Effect of rootstock and interstock on leaf macronutrients, micronutrients and heavy metals concentration.

As illustrated in **Figure 5** and detailed in **Table 10**, significant variability in macronutrient concentrations was observed among the different mango grafting combinations, reflecting the influence of rootstock and interstock on nutrient uptake and assimilation.The Mallika/Olour combination consistently demonstrated the highest concentrations of key macronutrients. Nitrogen content peaked at 1.86%, well above the overall mean of 1.22%, while the lowest value was recorded in Olour/Amrapali/Olour (0.70%). Similarly, phosphorus levels ranged from a high of 0.42% in Mallika/Olour to just 0.08% in Olour/Amrapali/Olour, indicating a pronounced disparity in phosphorus uptake efficiency. Calcium concentrations followed a comparable trend, with Mallika/Olour again leading at 3.02%, compared to the lowest value of 1.13% in Olour/Amrapali/Olour. Potassium content varied from 0.86% in Mallika/Olour to 0.41% in Olour/Olour. In contrast to the other nutrients, magnesium content was highest in Amrapali/Olour and Olour/Amrapali/Olour (both at 0.35%), and lowest in Olour/Mallika/Olour (0.16%). These results clearly indicate that the Mallika/Olour combination possesses a superior capacity for macronutrient absorption, supporting vigorous vegetative growth and efficient metabolic function. On the other hand, the Olour/Amrapali/Olour grafts consistently exhibited the lowest nutrient concentrations, suggesting limited nutrient uptake or internal transport efficiency.As presented in **Table 11**, leaf micronutrient concentrations varied significantly among the grafting combinations, indicating differential nutrient uptake and distribution influenced by rootstock–interstock interactions. The Mallika/Olour combination consistently exhibited superior micronutrient profiles. Zinc content reached its highest level in this treatment (47.43 ppm), while the lowest concentration (28.02 ppm) was found in Olour/Amrapali/Olour. Iron and manganese followed similar trends, peaking in Mallika/Olour at 161.21 ppm and 93.16 ppm, respectively, and reaching minimum values in Olour/Olour (58.68 ppm) and Olour/Amrapali/Olour (23.05 ppm). Copper levels deviated from this pattern, with the highest concentration observed in Olour/Olour (68.28 ppm) and the lowest in Olour/Amrapali/Olour (21.86 ppm). Boron content ranged from 48.03 ppm in Mallika/Olour to 23.21 ppm in Olour/Amrapali/Olour, reinforcing the trend of improved micronutrient accumulation in the Mallika-based grafts. Sodium concentration was notably highest in Amrapali/Olour (309.08 ppm) and lowest in Olour/Mallika/Olour (123.77 ppm), suggesting variation in ion regulation and possibly stress response mechanisms among treatments. These findings underscore the enhanced micronutrient uptake capacity of the Mallika/Olour combination, which likely supports critical physiological processes such as enzyme activation, chlorophyll synthesis, and stress resilience. In contrast, the consistently lower micronutrient levels observed in Olour/Amrapali/Olour suggest potential limitations in root function or nutrient translocation efficiency. Heavy metal accumulation in leaves (**Table 12**) further demonstrated the differential impact of grafting combinations. Cobalt concentrations ranged from 0.05 ppm in Olour/Amrapali/Olour and Olour/Mallika/Olour to 0.19 ppm in Olour/Olour. Aluminium levels were highest in Amrapali/Olour (51.86 ppm) and lowest in Olour/Mallika/Olour (12.22 ppm). Chromium content ranged from 1.29 ppm in Olour/Amrapali/Olour to 3.73 ppm in Olour/Olour, and nickel from 1.18 ppm in Olour/Amrapali/Olour to 5.46 ppm in Olour/Olour. Cadmium levels were highest in Amrapali/Olour (0.72 ppm) and lowest in Olour/Olour (0.13 ppm). Strontium and lead also followed a similar trend, with the highest concentrations in Amrapali/Olour (97.05 ppm and 1.71 ppm, respectively) and the lowest in Olour/Mallika/Olour (37.42 ppm and 0.73 ppm, respectively). These observations suggest that certain combinations, particularly Amrapali/Olour and Olour/Olour, may be more prone to translocating heavy metals to aerial parts. In contrast, the Mallika/Olour combination demonstrated limited heavy metal accumulation, indicating its effectiveness in restricting toxic element uptake and improving selectivity in nutrient absorption. **Figure 5** correlates with the results on mineral nutrient and heavy metal accumulation in leaves. It highlights that the Olour/Mallika/Olour combination not only facilitates better uptake of essential macro and micronutrients such as nitrogen, phosphorus, potassium, calcium, and magnesium but also shows the lowest accumulation of toxic heavy metals like aluminum and cobalt. This suggests superior selective absorption and nutrient utilization capacity, potentially may be due to improved root system architecture and physiological compatibility provided by the interstock. Conversely, the Amrapali/Olour combination, while enhancing nutrient content, showed elevated heavy metal levels, possibly indicating less efficient exclusion or detoxification mechanisms.

**Table 10:** Effect of rootstock and interstock on leaf macronutrients concentration.

Grafting combination	Nitrogen (%)	Phosphorus (%)	Calcium (%)	Potassium (%)	Magnesium (%)
O/O	1.38^b^	0.22^c^	1.80^c^	0.41^e^	0.28^b^
A/O	0.91^cd^	0.15^d^	2.21^b^	0.62^c^	0.35^a^
M/O	1.86^a^	0.42^a^	3.02^a^	0.86^a^	0.22^bc^
O/A/O	0.70^d^	0.08^e^	1.13^d^	0.54^d^	0.35^a^
O/M/O	1.24^bc^	0.34^b^	2.61^ab^	0.73^b^	0.16^c^
Mean	1.22	0.24	2.15	0.63	0.27
CV	6.01	9.29	5.83	3.54	5.55
SE (m) ±	0.03	0.01	0.06	0.01	0.06
LSD (p ≤ 0.05)	0.09	0.04	0.17	0.04	0.18

Superscript in small letters on the value of each parameter indicates significant difference at *p ≤ 0.05*.

**Table 11:** Effect of rootstock and interstock on leaf micronutrients concentration.

Grafting combination	Zinc (ppm)	Copper (ppm)	Iron (ppm)	Manganese (ppm)	Boron (ppm)	Sodium (ppm)
O/O	34.98^c^	68.28^a^	58.68^e^	35.57^c^	26.34^d^	207.88^c^
A/O	33.41^d^	32.22^d^	92.48^c^	27.03^d^	31.16^c^	309.08^a^
M/O	47.43^a^	45.11^b^	161.21^a^	93.16^a^	48.03^a^	171.93^d^
O/A/O	28.02^e^	21.86^e^	61.82^d^	23.05^e^	23.21^e^	223.49^b^
O/M/O	40.18^b^	42.21^c^	132.77^b^	55.81^b^	36.14^b^	123.77^e^
Mean	36.80	41.94	101.39	46.92	32.97	207.30
CV	3.19	4.53	2.00	6.35	4.69	4.42
SE (m) ±	0.52	0.85	0.91	1.33	0.69	0.40
LSD (p ≤ 0.05)	1.57	2.60	2.74	3.97	2.08	1.23

Superscript in small letters on the value of each parameter indicates significant difference at *p ≤ 0.05*.

**Table 12:** Effect of rootstock and interstock on leaf heavy metals concentration.

Grafting combination	Cobalt (ppm)	Aluminium (ppm)	Chromium (ppm)	Nickel (ppm)	Cadmium (ppm)	Strontium (ppm)	Lead (ppm)
O/O	0.19^a^	26.17^c^	3.73^a^	5.46^a^	0.13^d^	73.37^c^	1.55^b^
A/O	0.07^b^	51.86^a^	1.51^c^	1.29^c^	0.72^a^	97.05^a^	1.71^a^
M/O	0.08^b^	17.90^d^	2.89^b^	2.38^b^	0.28^c^	47.60^d^	0.95^d^
O/A/O	0.05^c^	25.05^c^	1.29^c^	1.18^c^	0.42^b^	92.18^ab^	1.14^c^
O/M/O	0.05^c^	12.22^e^	1.88^b^	1.49^c^	0.21^cd^	37.42^e^	0.73^e^
Mean	0.09	26.64	2.26	2.36	0.37	69.53	1.22
CV	12.14	6.37	7.89	12.16	7.33	3.57	9.14
SE (m) ±	0.01	0.75	0.08	0.12	0.01	1.11	0.05
LSD (p ≤ 0.05)	0.03	2.27	0.24	0.36	0.04	3.33	0.16

Superscript in small letters on the value of each parameter indicates significant difference at *p ≤ 0.05*.

## Discussion

### Physical growth parameters

The significant variations in rootstock girth, scion girth, stock-to-scion ratio, and internodal length among different grafting combinations highlight the influence of stock-scion compatibility on vegetative growth. The superior performance of the Mallika/Olourcombination in terms of rootstock girth (10.28 mm) and scion girth (6.33 mm) suggests enhanced vigour, likely due to efficient nutrient uptake and water transport facilitated by the rootstock’s robust vascular connections (Perez et al., 1993). Additionally, the relatively consistent stock-to-scion ratio across combinations could suggest strong graft compatibility, a critical factor for sustained growth and productivity (Hartmann et al., 2002). The greater internodal length in the Mallika/Olour combination (1.670 cm) further corroborates its superior vegetative potential, consistent with findings by Perez et al. (1993). This is supported by recent studies indicating that compatible graft combinations, often those with superior girth development, also exhibit increased internodal length, suggesting more vigorous shoot growth (Ismail and Ebeed, 2013). Studies have demonstrated that different rootstock genotypes significantly influence scion vigour and tree architecture in mango (Ali, 2023).

### Leaf morphological and physiological traits

The observed variability in leaf morphological traits, such as leaf length, width, fresh weight, and leaf area, underscores the role of rootstock and interstock in determining the photosynthetic and assimilatory potential of grafted plants. The Mallika/Olour combination, exhibiting the highest leaf area (56.48 cm²) and fresh weight (3.03 g), aligns with reports by Perez et al. (1988) which associate larger leaf areas with higher photosynthetic efficiency and biomass accumulation. Furthermore, the superior physiological traits in Mallika/Olour, such as stomatal conductance (0.070 mol m^-2^ s^-1^) and net photosynthesis (8.51 μmol m^-2^ s^-1^), emphasize its potential for enhanced carbon assimilation. These results align with earlier studies indicating that rootstock-scion combinations significantly influence gas exchange and water use efficiency in fruit crops (Chauhan, 2006; Perez et al., 1993). It has been shown that photosynthesis variables, including stomatal conductance, intercellular CO_2_ concentration, and transpiration, were all significantly influencedby the rootstock (Koepke and Dhingra, 2013). Losciale et al. (2008) found that ‘Bosc’ pear grafted onto quince EMC rootstocks allocated fewer photons to photosynthesis, increasing light dissipation and PSII damage under stress. Size-controlling rootstocks, with reduced light absorption and limited CO_2_ assimilation under water stress, are more prone to photo-oxidative damage, ultimately reducing photosynthetic efficiency. Similarly, Minja et al. (2017) observed that Alphonso grafted onto Ngwangwa rootstock exhibited the greatest leaf development—recording the highest number of leaves (3.17), longest leaf length (16.83 cm), and greatest leaf width (24.42 cm) - four months post-grafting, further affirming the critical role of rootstock in influencing early vegetative growth and leaf morphology. Seedling evaluations highlight the critical role of genotype in vegetative vigour and physiological traits. Abirami et al. (2011) found that among polyembryonic mango genotypes, ‘Nekkare’ showed the highest seedling height, vigour, and leaf area, while in monoembryonic types, Amrapali recorded the lowest vigour and leaf area. Dayal et al. (2016) further reinforced the physiological impact of polyembryonic rootstocks, reporting that Olour rootstock increased total chlorophyll content and photosynthetic rate in Pusa Surya, Mallika, and Dashehari, while K-5 enhanced photosynthesis in Amrapali and Dashehari. Additionally, Olour improved transpiration rates in Pusa Arunima, Pusa Surya, and Amrapali. According to Hartmann et al. (2011), dwarfing rootstocks can modulate key physiological parameters including photosynthetic rate, transpiration rate, stomatal conductance, and osmotic potential. Similarly, Zhou et al. (2021) observed that the use of dwarfing interstocks in ‘Red Fuji’ apple scions leads to a downregulation of photosynthetic capacity. Srivastav et al. (2009) examined the association between physiological traits and vigour indices in 16 polyembryonic mango genotypes. Their findings identified chlorophyll ‘a’ content, net CO_2_ assimilation rate, and total leaf phenol content as key physiological markers for evaluating seedling vigour at the nursery stage.

### Biochemical composition

Biochemical analysis revealed significant differences in protein, sugar, phenol, and proline content across grafting combinations. The highest total soluble protein (4.34 mg/g FW) and total sugars (119.05 mg/g FW) in the Olour/Mallika/Olourcombination could suggest enhanced photosynthetic efficiency (showing high leaf net photosynthesis) and nutrient assimilation, consistent with findings by Sampaio and Simao (1996). Elevated levels of phenols (3067.53 mg/100 g) and proline (1.06 μg/g FW) in the Olour/Amrapali/Olour combination could suggest a physiological orientation towards growth regulation, which may contribute to its dwarfing nature. These compounds are known to play critical roles in modulating cell division, lignification, and osmotic adjustment, processes that, while enhancing stress resilience, can also result in restricted vegetative growth and reduced vigour (Yong et al., 1983). These variations reflect the metabolic adaptations imparted by rootstock and interstock combinations, crucial for optimizing performance under varying environmental conditions. Iyer (1991) suggested that higher phenolic concentrations in the apical bud correlate with reduced vigour and dwarfing in mango which are in line with the findings. Earlier, Hayat et al. (2022) reported that rootstocks significantly influence the soluble protein content in scion trees of ‘Shatangju’ mandarin. Similarly, Satisha et al. (2007) observed a notable impact of rootstocks on leaf protein content in grapevines.Phenolic compounds are abundantly present in the bark and leaves of fruit trees. Studies on apple rootstocks have indicated a link between bark phenols and indole-3-acetic acid (IAA) metabolism. Metabolomic analysis by Cui et al. (2021) revealed that the use of ‘OHF51’ (‘Old Home’ × ‘Farmingdale’) as an interstock altered metabolite profiles in both scion and rootstock, with phenolic acids and their derivatives identified as key contributors to scion dwarfism. Interstocks can induce physiological and biochemical changes in the scion. Fayek et al. (2022) reported that Paulsen 1103, used as an interstock with Flame Seedless grape under drought stress, enhanced leaf proline, phenol, and total sugar content, resulting in notable physiological and anatomical modifications in the scion. Sridevi et al. (2021) assessed physiological and biochemical traits in eight mango half-sib populations at IIHR, Bengaluru. Goa Mankurad showed the highest phenol content (197.81 mg/100 g FW).

### Photosynthetic pigments and stomatal anatomy

The significant differences in photosynthetic pigments across combinations, with Olour/Mallika/Olourexhibiting the highest chlorophyll A (3.07 mg/g FW) and total chlorophyll (4.04 mg/g FW) contents, highlight its superior capacity for light harvesting and energy transfer (Feungchan et al., 1992). The chlorophyll A:B ratio, ranging from 2.41 (Olour/Amrapali/Olour) to 3.32 (Olour/Mallika/Olour), indicates variations in the efficiency of photosystem II, consistent with findings by Avila et al., 1993. These findings align with previous studies emphasizing the value of photosynthetic pigments. Abirami et al. (2011) identified chlorophyll fractions, along with phenolic content, as reliable predictors of rootstock vigour at the seedling stage. Further supporting this, Abou-Ellail et al. (2014) demonstrated wide genetic variability in Egyptian mango genotypes, with chlorophyll content ranging from 202.5 µg/g to 386.9 µg/g and carotenoid content from 19.9 µg/g to 106.2 µg/g. These results align with those of Dayal et al. (2016), who reported that the Olour rootstock induced the highest total chlorophyll content in cultivars Pusa Surya, Mallika, and Dashehari, reinforcing its role in improving pigment concentration and photosynthetic performance. Deepak et al. (2017) assessed three polyembryonic mango rootstocks - Olour, Vellaikolumban, and Nekkare - and found that Nekkare had the highest chlorophyll a, b, and total chlorophyll content, classifying it as a vigorous rootstock. Olour showed intermediate chlorophyll levels (semi-vigorous), while Vellaikolumban had the lowest, indicating the least vigour. Similarly, Mog and Nayak (2018) evaluated fifteen cashew accessions based on photosynthetic pigments and reported significant histological differences. ‘Bhaskara’ exhibited the highest chlorophyll a and total chlorophyll content, whereas ‘Selection-2’ recorded the highest carotenoid concentration, highlighting the role of pigment traits in assessing vigour. The anatomical analysis further revealed larger stomatal dimensions in the Mallika/Olour combination, including pore area (5708.57 μm²), which may facilitate enhanced gas exchange and transpiration. These results support the hypothesis that stomatal traits are modulated by rootstock-scion interactions, influencing water use efficiency and photosynthetic capacity (Hartmann et al., 2002; Mukherjee and Das, 1980). Stomatal density has been closely associated with plant vigour in mango.In mango, Chakladar (1967) and Bang (1970) reported a positive correlation between stomatal density and plant vigour, with vigorous rootstocks exhibiting higher stomatal densities. Similar associations were observed in apple (Beakbane and Majumder, 1975) and plum (Pathak et al., 1977), as well as in guava (Saroj et al., 1997). Majumder et al. (1972, 1981) even proposed a classification system for mango rootstocks based on stomatal traits.Earlier Zhou et al. (2020) also reported variation in stomatal density in scion varieties of apple and found more stomatal density in dwarfing rootstocks M.9 and B.9 than in vigorousrootstock Baleng on apple scion variety ‘Red Fuji’. Sridevi et al. (2021) reported that Goa Mankurad showed the highest stomatal density (1244/mm²), Malanji recorded the largest stomatal size (252.16 µm), and Mandoor Khatta exhibited the highest total chlorophyll content (6.69 mg/100 g FW).

### Nutrient uptake and heavy metal accumulation

The superior macronutrient uptake in the Mallika/Olour combination, particularly nitrogen (1.86%) and calcium (3.01%), underscores its enhanced ability to support growth and metabolic processes. The higher micronutrient levels, such as zinc (47.43 ppm) and iron (161.20 ppm), further indicate the role of rootstock in improving nutrient assimilation, consistent with earlier findings (Willis and Marler, 1993). Conversely, the Olour/Amrapali/Olour combination exhibited lower nutrient concentrations and higher heavy metal accumulation, reflecting reduced nutrient uptake efficiency and potential environmental stress susceptibility. The ability of the Mallika/Olour combination to limit heavy metal translocation to leaves aligns with studies by Jones (1984), emphasizing its potential for sustainable cultivation in contaminated soils. These findings are consistent with extensive research across perennial fruit crops, which has established that rootstocks play a pivotal role in modulating mineral uptake, transport, and assimilation. In apple (*Malus domestica*), rootstocks such as M9 and MM106 exhibit distinct efficiencies in the absorption of key nutrients, including nitrogen (N), potassium (K), calcium (Ca), phosphorus (P), manganese (Mn), and iron (Fe) (Amiri et al., 2014). Similarly, in grapevine (*Vitis vinifera*), rootstock architecture significantly influences nitrogen use efficiency (NUE) and phosphorus acquisition (Zamboni et al., 2016). In stone fruit species, rootstocks have been shown to affect the macro and micronutrient composition of vegetative and reproductive tissues, as reported in cherry (*Prunus avium*) (Hrotko et al., 2014; Jimenez et al., 2004), peach (*Prunus persica*) (Mestre et al., 2015; Zarrouk et al., 2005), and plum (*Prunus domestica*) (Reig et al., 2018), emphasizing the central role of rootstocks in nutrient dynamics across diverse fruit crops. Lazare et al. (2020) demonstrated that avocado rootstocks influence tree nutritional status through differential mineral transport, as reflected in the leaf ionome of the scion. Rossdeutsch et al. (2020) found that in grapevines, less vigorous rootstocks exhibited greater nitrate uptake and assimilation following nitrate resupply compared to more vigorous rootstocks, likely due to higher root carbohydrate reserves. Sarkhosh et al. (2021) explored the influence of rootstocks on scion nutrient status and highlighted their role in cultivar improvement.

### Multivariate analysis

**Figure 6:**
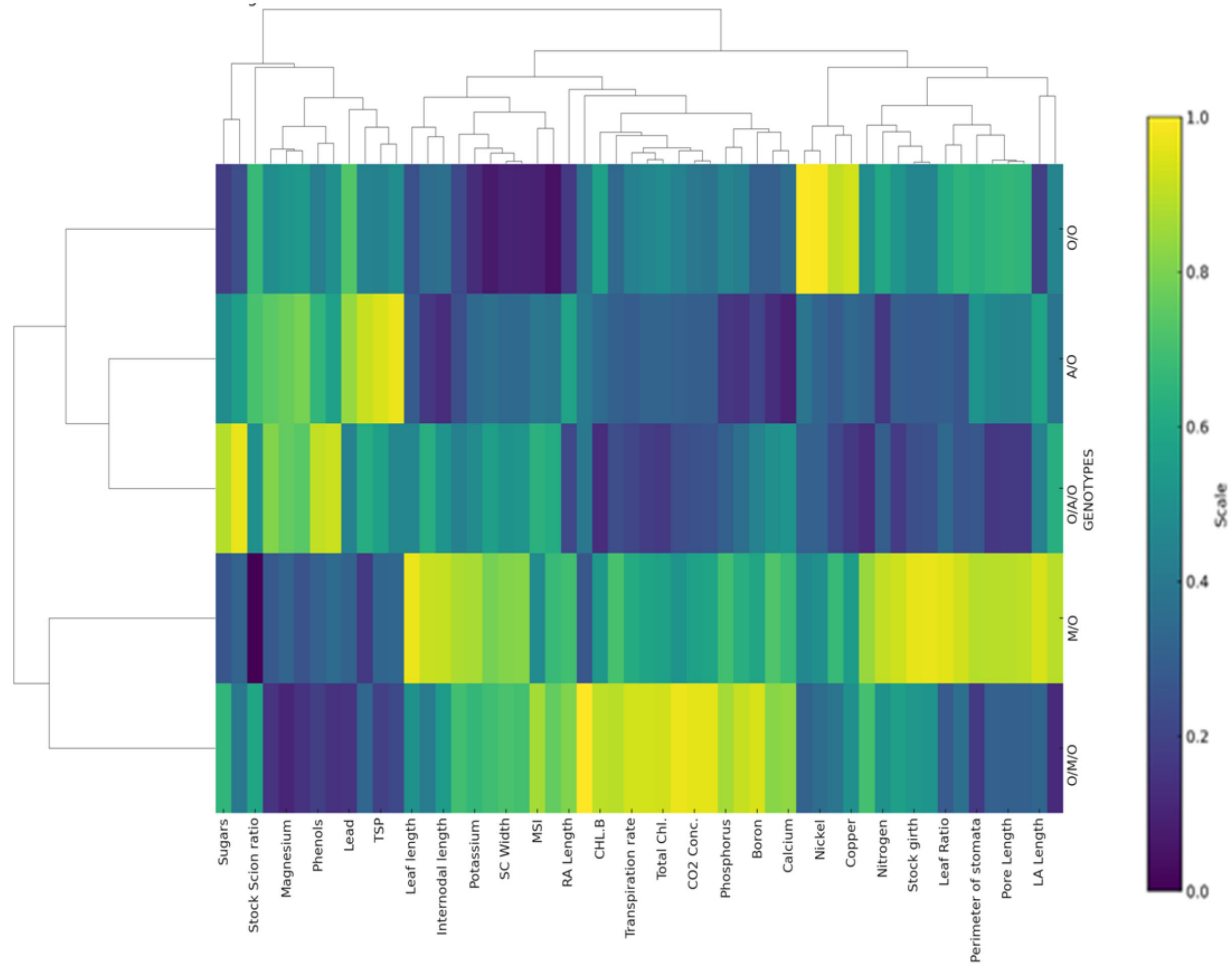
Heat map and hierarchical clustering of all grafting combinations for morpho-physio-chemical, nutrient contents, and anatomical parameters.

The heat map and hierarchical clustering analysis (**Figure 6**) revealed distinct groupings of grafting combinations based on morpho-physio-chemical, nutrient contents, and anatomical parameters. Each row represents a specific grafting combination, while each column corresponds to a measured parameter, such as sugars, stomatal characteristics, chlorophyll content, mineral nutrients, etc. The colour scale reflects the relative magnitude of each parameter, with dark blue or purple shades indicating lower values, green representing intermediate values, and yellow denoting higher values. This colour gradient enables quick visual identification of which combinations performed better or worse for each trait.Grafting combinations with similar traits were clustered together, indicating shared physiological and biochemical attributes. For instance, combinations such as Olour/Mallika/Olour and Mallika/Olour were closely grouped due to their higher total protein and sugar contents, while Amrapali/Olour and Olour/Amrapali/Olour formed a separate cluster characterized by elevated phenol and proline levels. This clustering reflects the influence of rootstock and interstock combinations on the overall biochemical, physiological and anatomical profiles, suggesting that specific grafting combination scan be targeted to optimize desired traits. These findings support earlier studies emphasizing the role of grafting in modulating biochemical profiles (Hayat et al., 2022; Cui et al., 2021; Fayek et al., 2022; Yong et al., 1983).

**Figure 7:**
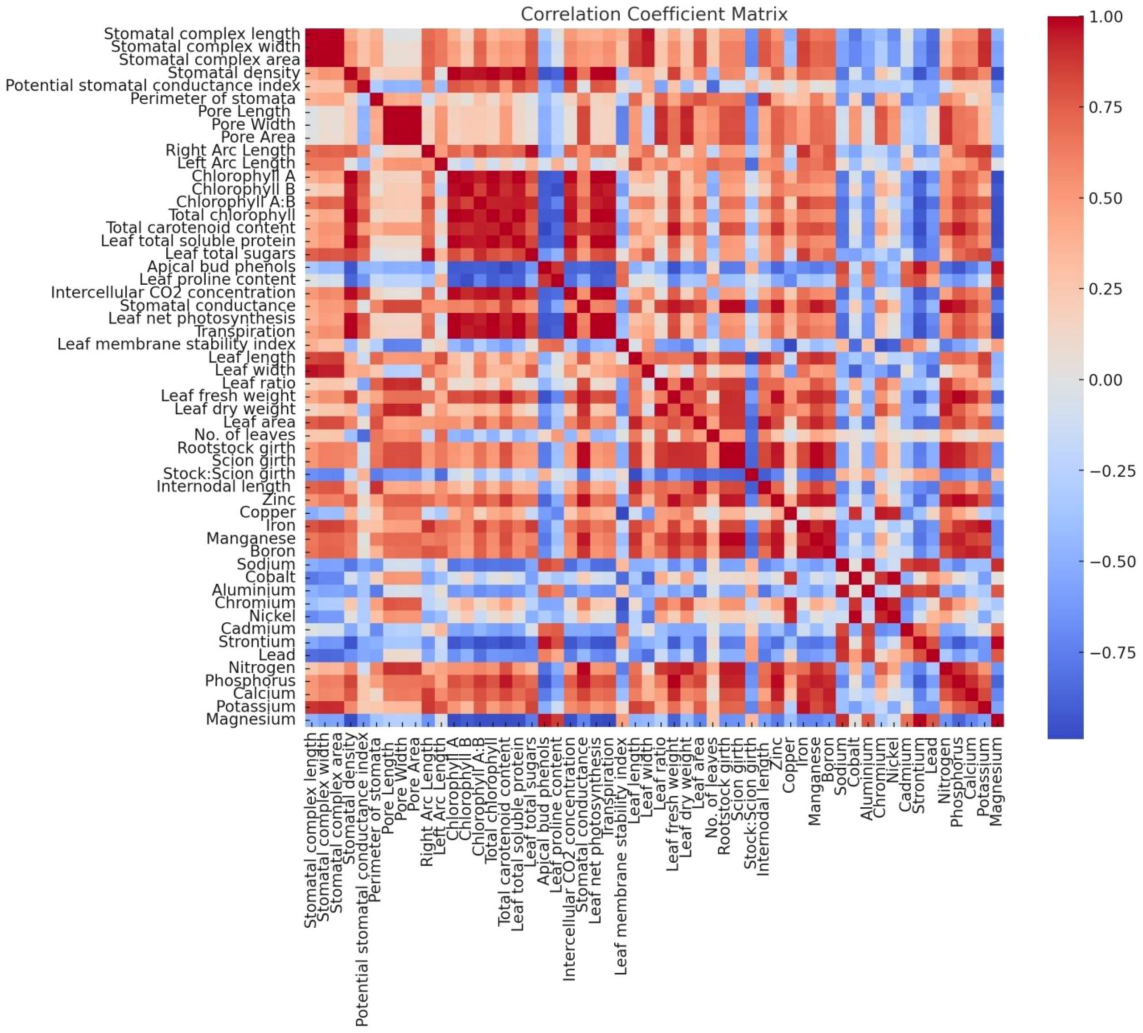
Pearson’s Correlation matrix for morpho-physio-chemical, nutrient contents, and anatomical parameters.

The correlation analysis highlighted significant relationships among the measured parameters (**Figure 7**). Leaf total soluble protein showed a strong positive correlation with total sugars (r > 0.8, p< 0.05), suggesting that protein synthesis may be linked to carbohydrate metabolism in grafted plants. Conversely, phenol content exhibited a negative correlation with total sugars and protein, indicating a trade-off between primary and secondary metabolite pathways, consistent with the findings of Cui et al. (2021); Satisha et al. (2007) and Yong et al. (1983), who observed similar metabolic adjustments in grafted plants. Proline content was positively correlated with phenols but negatively correlated with protein and sugars, reflecting its role in vigour control and osmoprotection under variable physiological conditions. These correlations highlight the complex interplay of metabolic pathways influenced by grafting combinations.

**Figure 8:**
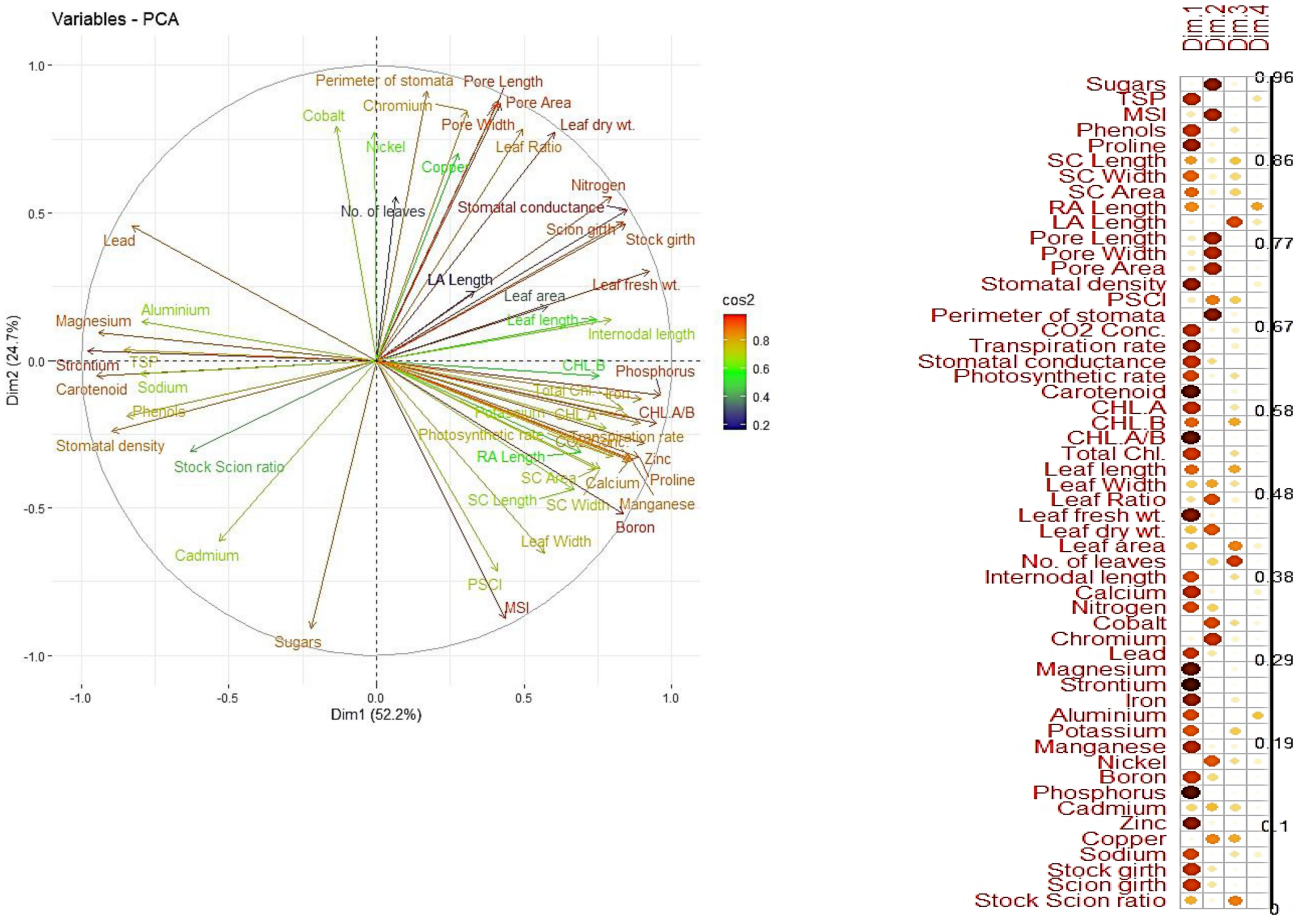
Principal component analysis showing dispersion of grafting combinations based on measured parameters.

The principal component analysis (PCA) revealed that the first two principal components (PC1 and PC2) accounted for the majority of the variance in the dataset, cumulatively explaining over 80% of the variability (**Figure 8**). PC1 was primarily associated with parameters like total protein, sugars, and phenols, while PC2 was strongly influenced by proline and anatomical traits. Vigorous combinations like Olour/Mallika/Olour were positioned prominently in the PCA biplot, associated with traits favouring photosynthetic efficiency and metabolic vigour. In contrast, Olour/Amrapali/Olour exhibited a clear separation based on traits related to dwarfness, including higher proline and phenol levels, which contribute to stress tolerance and reduced vegetative growth. These results indicate that rootstock and interstock combinations contribute significantly to the phenotypic and metabolic diversity in grafted plants, allowing the identification of specific combinations for tailored traits, as supported by earlier findings (Yonemoto et al., 2007).

These results align with existing research showing that grafting combinations influence tree size, nutrient uptake efficiency, and stress responses (Karlidag et al., 2014). Vigorous interstocks, such as those used in the Olour/Mallika/Olour combination, promote faster canopy development and efficient nutrient utilization, which are essential for maximizing light interception and photosynthetic output. In contrast, dwarfing combinations like Olour/Amrapali/Olour are advantageous for high-density planting systems, as their compact growth habit facilitates management and maximizes space utilization (Yonemoto et al., 2007).

The integration of heat map clustering, correlation analysis, and PCA underscores the multidimensional nature of grafting-induced variations in plants. The clustering results align with the observed biochemical trends, confirming that rootstock and interstock combinations influence nutrient allocation and metabolic adjustments. Correlation analysis highlights the interdependence of key physiological pathways, such as the trade-offs between primary and secondary metabolites, which are crucial for optimizing plant performance under different environmental and nutritional conditions. PCA further strengthens this understanding by visually separating grafting combinations based on their unique trait profiles, providing a robust framework for selecting combinations tailored to specific traits. These findings emphasize the importance of multivariate approaches in understanding the complex interactions that govern plant responses to grafting.

## Conclusion

This study underscores the critical influence of rootstock and interstock combinations on nutrient uptake, vegetative growth, and physiological efficiency in mango cultivation. Among the evaluated combinations, Olour/Mallika/Olour demonstrated strong potential for promoting vigour, characterized by balanced nutrient uptake and reduced accumulation of heavy metals. Its ability to support robust growth through efficient assimilation of macro- and micronutrients underscores its physiological efficiency and overall plant health. The enhanced vegetative performance associated with this combination also supports rapid canopy development, a key factor in improving light interception and fruit-bearing efficiency.In contrast, the Olour/Amrapali/Olour combination exhibited traits associated with dwarfing, including lower nutrient uptake and restricted growth. This makes it a suitable candidate for high-density planting systems, where compact tree structure is essential for ease of management and space optimization. The contrasting growth responses between Olour/Mallika/Olour and Olour/Amrapali/Olour emphasize the importance of selecting appropriate grafting combinations to align with specific production objectives.These findings provide valuable direction for the development of sustainable mango production strategies. By selecting suitable rootstock and interstock combinations, growers can tailor orchard design and management practices to optimize yield, resource use, and plant health. Further long-term field evaluations under diverse agro-climatic conditions are recommended to confirm the performance and adaptability of these grafting combinations.

## Data Availability

The original contributions presented in the study are included in the article/supplementary material. Further inquiries can be directed to the corresponding author.

## References

[B1] Abirami K. Singh R. Bhaskaran V. (2011). Studies on the influence of seedling physiological parameters with vigour in some polyembryonic and monoembryonic mango genotypes. Indian J. Horticult. 68, 18–23.

[B2] Abou-Ellail M. El-Shabrawi H. M. Matter M. A. Aly U. I. Ghareeb H. A. Eissa E. A. (2014). Appraisal of biochemical and genetic diversity of mango cultivars using molecular markers. Afr. J. Biotechnol. 13, 2796–2806. doi: 10.5897/AJB2014.13685

[B3] Ahmed W. Nawaz M. A. Iqbal M. A. Khan M. (2007). Effect of different rootstocks on plant nutrient status and yield in Kinnow mandarin (*Citrus reticulata* Blanco). Pak. J. Bot. 39, 1779–1786.

[B4] Ali A. (2023). Influence of different rootstocks on growth, yield and fruit quality of mango (*Mangifera indica* L.) cv. Dashehari. J. Appl. Horticult. 25, 60–65.

[B5] Amiri M. E. Fallahi E. Safi-Songhorabad M. (2014). Influence of rootstock on mineral uptake and scion growth of ‘Golden delicious’ and ‘Royal Gala’ apples. J. Plant Nutr. 37, 16–29.

[B6] Anonymous (2023). Area and Production of Horticulture Crops (2^nd^ Advance Estimate) (India: Ministry of Agriculture and Farmers Welfare. Government of India, Directorate of Economics and Statistics).

[B7] Avila C. R. Garcia E. P. Matheis L. T. Mosqueda R. V. (1993). Production efficiency of compact ‘Manila’ mangoes grafted onto different interstock-rootstock. Acta Hortic., 281–287.

[B8] Bang A. (1970). “Nursery selection of mango rootstocks,” in M.Sc. (Agri.) thesis(lARI, New Delhi).

[B9] Bates L. S. Waldren R. P. Teare I. D. (1973). Rapid determination of free proline for water-stress studies. Plant Soil 39, 205–207. doi: 10.1007/BF00018060

[B10] Bavaresco L. Giachino E. Pezzutto S. (2003). Grapevine rootstock effects on lime-induced chlorosis, nutrient uptake, and source–sink relationships. J. Plant Nutr. 26, 1451–1465. doi: 10.1081/PLN-120021054

[B11] Baxter I. R. Vitek O. Lahner B. Muthukumar B. Borghi M. Morrissey J.. (2008). The leaf ionome as a multivariable system to detect a plant’s physiological status. Proc. Natl. Acad. Sci. 105, 12081–12086. doi: 10.1073/pnas.0804175105, PMID: 18697928 PMC2515221

[B12] Beakbane A. B. Majumder P. K. (1975). A relationship between stomatal density and growth potential in apple rootstocks. J. Hortic. Sci. 50, 285–289.

[B13] Bompard J. M. (2009). “Taxonomyandsystematics,” in The mango: Botany, Production and Uses. Ed. Litz R. E. (CABI, Cambridge), 19–41.

[B14] Bosa K. Jadczuk-Tobjasz E. Kalaji M. Majewska M. Allakhverdiev S. (2014). Evaluating the effect of rootstocks and potassium level on photosynthetic productivity and yield of pear trees. Russ. J. Plant Physiol. 61, 231–237. doi: 10.1134/S1021443714020022

[B15] Bradford M. M. (1976). A rapid and sensitive method for the quantitation of microgramquantities of protein utilizing the principle of protein-dye binding. Anal. Biochem. 72, 248 254., PMID: 942051 10.1016/0003-2697(76)90527-3

[B16] Chakladar B. P. (1967). “Selection and classification of mango rootstocks in the nursery stage,” in M.Sc. (Hort.) thesis(lARI, New Delhi).

[B17] Chauhan J. S. (2006). Studies on the effect of rootstock and interstock on morphological, physiological and anatomical characteristics of pears (Nauni, Solan (HP: Dr YS Parmer University of Horticulture and Forestry).

[B18] Cheng L. Raba R. (2009). Accumulation of macro-and micronutrients and nitrogen demand-supply relationship of ‘Gala’/’Malling 26’apple trees grown in sand culture. J. Am. Soc Hortic. Sci. 134, 3–13. doi: 10.21273/JASHS.134.1.3

[B19] Cordeiro M. C. R. Pinto A. C. Q. Ramos V. H. V. Faleiro F. G. Fraga L. M. S. (2006). Identification of plantlet genetic origin in polyembryonic mango (*Mangifera indica* L.) cv. Rosinha seeds using RAPD markers. RevistaBrasileira Fruticult. 28, 454–457.

[B20] Costes E. García-Villanueva E. (2007). Clarifying the effects of dwarfing rootstock on vegetative and reproductive growth during tree development: a study on apple trees. Ann. Bot. 100, 347–357. doi: 10.1093/aob/mcm114, PMID: 17652339 PMC2735327

[B21] Costes E. Sinoquet H. Kelner J. J. Godin C. (2003). Exploring within tree architectural development of two apple tree cultivars over 6 years. Ann. Bot. 91, 91–104. doi: 10.1093/aob/mcg010, PMID: 12495924 PMC4240355

[B22] Cui Z. Zhang H. Galarneau E. R. A. (2021). Metabolome analysis reveals important compounds related to dwarfing effect of interstock on scion in pear. Ann. Appl. Biol. 179, 108–122. doi: 10.1111/aab.12684

[B23] Davis A. R. Perkins-Veazie P. Sakata Y. Lopez-Galarza S. Maroto J. V. (2008). Cucurbitgrafting. Crit. Rev. Plant Sci. 27, 50–74. doi: 10.1080/07352680802053940

[B24] Dayal V. Dubey A. Singh S. Sharma R. Dahuja A. Kaur C. (2016). Growth, yield and physiology of mango (*Mangifera indica* L.) cultivars as affected by polyembryonic rootstocks. Sci. Hortic. 199, 186–197. doi: 10.1016/j.scienta.2015.12.042

[B25] Deepak G. N. Jeevan U. Priyanka H. Bhagya H. Jaganath S. (2017). Vegetative performance of polyembryonic mango (*Mangifera indica* L.) rootstocks under Eastern dry zone of Karnataka. Int. J. Curr. Microbiol. Appl. Sci. 20, 595–597.

[B26] Dubey A. K. Srivatsav M. Sharma Y. K. Pandey R. N. Deshmukh P. S. (2007). Dry mass production and distribution of nutrients in two mango rootstocks as affected by salinity. Indian J. Horticult. 64, 385–390.

[B27] Fallahi E. Chun I. J. Neilsen G. H. Colt W. M. (2001). Effects of three rootstocks on photosynthesis, leaf mineral nutrition, and vegetative growth of “BC-2 Fuji” apple trees. J. Plant Nutr. 24, 827–834. doi: 10.1081/PLN-100103776

[B28] Fayek M. A. Ali A. E. Rashedy A. A. (2022). Physiological and chemical performance of the Flame seedless grapevine cultivar in the presence of Paulsen 1103 as the interstock. Ciencia e Agrotecnol. 46. doi: 10.1590/1413-7054202246021621

[B29] Feungchan S. Yimsawat T. Chindaprasert S. (1992). Effect of interstock ofmango cv. Monkom on growth of KhiewSawauy mango. KaenKasetKhonKaen. Agric. J. 20, 198–201.

[B30] Gonçalves B. Santos A. Silva A. P. Moutinho-Pereira J. Torres-Pereira J. M. G. (2003). Effect of pruning and plant spacing on the growth of cherry rootstocks and their influence on stem water potential of sweet cherry trees. J. Hortic. Sci. Biotech. 78, 667–672.

[B31] Haak E. Kviklys D. Lepsis J. (2006). Comparison of cydonia and pyrus rootstocks in Estonia, Latvia and Lithuania. Sodininkysteirdarzininkyste 25, 322–326.

[B32] Habran A. Commisso M. Helwi P. (2016). Roostocks/scion/nitrogen interactions affect secondary metabolism in the grape berry. Front. Plant Sci. 7, 1134. doi: 10.3389/fpls.2016.01134, PMID: 27555847 PMC4977291

[B33] Haroldsen V. M. Chi-Ham C. L. Bennett A. B. (2012). Transgene mobilization and regulatory uncertainty for non-GE fruit products of transgenic rootstocks. J. Biotechnol. 161, 349–353. doi: 10.1016/j.jbiotec.2012.06.017, PMID: 22749907

[B34] Hartmann H. T. Kester D. E. Davies F. T. Geneve R. L. (2002). Plant Propagation: Principles and Practices (7^th^ ed.) (Upper Saddle River, NJ: Pearson Education).

[B35] Hartmann H. T. Kester D. E. Geneve (2011). Hartmann & Kester’s Plant Propagation Principles and Practices, Upper Saddle River (Nueva Jersey, Estados Unidos). 8th (Nueva Jersey, Estados Unidos, US State: Prentice Hall), 915.

[B36] Hayat F. Li J. Liu W. Li C. Song W. Iqbal S. (2022). Influence of citrus rootstocks on scion growth, hormone levels, and metabolites profile of “Shatangu. Mandarin. Hortic. 8, 608.

[B37] Hiscox J. D. Israelstam G. F. (1979). Amethodfortheextractionof chlorophyll from leaf tissue without maceration. Canad. J. Bot. 57, 1332–1334. doi: 10.1139/b79-163

[B38] Hrotko K. Magyar L. Borsos G. Gyeviki M. (2014). Rootstock effect on nutrient concentration of sweet cherry leaves. J. Plant Nutr. 37, 1395–1409. doi: 10.1080/01904167.2014.911317

[B39] Ismail M. O. Ebeed S. (2013). Evaluation of some mango interstock on ‘Kiet’Scion growth. J. Adv. Biol. 3, 201–203.

[B40] Iyer C. P. A. (1991). Recent advances in varietal improvement in mango. Acta Hortic. 291, 109–132. doi: 10.17660/ActaHortic.1991.291.14

[B41] Jackson M. L. (1973). Soilchemicalanalysis (New Delhi: Prentice Hall of Indian Private Limited), p 498.

[B42] Jimenez S. Garin A. Gogorcena Y. (2004). Flower and foliar analysis for prognosis of sweet cherry nutrition: influence of different rootstocks. J. Plant Nutr. 27, 701–712. doi: 10.1081/PLN-120030376

[B43] Jiménez S. Pinochet J. Gogorcena Y. Betrán J. Moreno M. (2007). Influence of different vigour cherry rootstocks on leaves and shoots mineral composition. Sci. Hortic. 112, 73–79. doi: 10.1016/j.scienta.2006.12.010

[B44] Jones P. O. (1984). Modeofactionofrootstock/scioninteractionsinappleandcherry. Acta Hortic. 146, 175–182. doi: 10.17660/ActaHortic.1984.146.19

[B45] Karlidag H. Aslantaş R. Eşitken A. (2014). Effects of interstock (M9) length grafted ontoMM106 rootstock on sylleptic shoot formation, growth and yield in some apple cultivars. J. Agric. Sci. 20, 331–336.

[B46] Koepke T. Dhingra A. (2013). Rootstock scion somatogenetic interactions in perennial composite plants. Plant Cell Rep. 2, 1321–1337. doi: 10.1007/s00299-013-1471-9, PMID: 23793453 PMC4244527

[B47] Lazare S. Haberman A. Yermiyahu U. Erel R. Simenski E. Dag A. (2020). Avocado rootstock influences scion leaf mineral content. Arch. Agron. Soil Sci. 66, 1399–1409. doi: 10.1080/03650340.2019.1672163

[B48] Loreti F. Massai R. Fei C. Cinelli F. (2002). Performance of” Conference” cultivar on several quince and pear rootstocks: preliminary results. Acta Hortic. 596, 311–318. doi: 10.17660/ActaHortic.2002.596.48

[B49] Losciale P. Zibordi M. Manfrini L. Grappadelli L. C. (2008). Effects of rootstock on pear photosynthetic efficiency. Acta Hortic. 800, 241–248. doi: 10.17660/ActaHortic.2008.800.28

[B50] Majumder P. K. Chakladar B. P. Mukerjee S. K. (1972). Selection and classification of mango rootstocks in the nursery stage. Acta Hortic. 24, 101–106. doi: 10.17660/ActaHortic.1972.24.17

[B51] Majumder P. K. Sharma D. K. Singh R. N. (1981). “Breeding for dwarfness in mango (*Mangifera indica* L.),” in National Symposium on Tropical and Subtropical fruit crops (Horticultural Society of India, Bangalore), 3.

[B52] Majumder P. K. Sharma D. K. Singh R. N. (1982). A study on high density orcharding in mango (*Mangifera indica* L. var. Amrapali). Punjab Hortic. J. 22, 123–127.

[B53] Meister R. Rajani M. Ruzicka D. Schachtman D. P. (2014). Challenges of modifying root traits in crops for agriculture. Trends Plant Sci. 19, 779–788. doi: 10.1016/j.tplants.2014.08.005, PMID: 25239776

[B54] Mestre L. Reig G. Betran J. A. (2015). Influence of peach–almond hybrids and plum-based rootstocks on mineral nutrition and yield characteristics of ‘big top’ nectarine in replant and heavy-calcareous soil conditions. Sci. Hortic. 192, 475–481. doi: 10.1016/j.scienta.2015.05.020

[B55] Minja R. R. Kimaro A. A. Mpanda M. Moshy S. Mwaijande V. Ngereza A.. (2017). Effects of rootstock type and scion cultivar on grafting success and growth of mango (*Mangifera indica* L.) seedlings. JEAI. 16, 1–9. doi: 10.9734/JEAI/2017/32129

[B56] Mog B. Nayak M. G. (2018). Leaf morphological and physiological traits and their significance in yield improvement of fifteen cashew varieties in West Coast Region of Karnataka. Int. J. Curr. Microbiol. Appl. Sci. 7, 1455–1469. doi: 10.20546/ijcmas.2018.707.173

[B57] Mukherjee S. K. (1951). Origin of mango. Indian J. Genet. 11, 49–56.

[B58] Mukherjee S. K. Das D. (1980). Anatomical screening of mango (*Mangifera indica*)seedlings for use as dwarfing rootstock. Sci. Cult. 46, 333–336.

[B59] Nawaz M. A. Imtiaz M. Kong Q. (2016). Grafting: a technique to modify ion accumulation in horticultural crops. Front. Plant Sci. 7, 1457. doi: 10.3389/fpls.2016.01457, PMID: 27818663 PMC5073839

[B60] North M. Cook N. (2006). “Effect of six rootstocks on ‘Forelle’pear tree growth, production, fruit quality and leaf mineral content,” in XXVII international horticultural congress-IHC2006: international symposium on enhancing economic and environmental, vol. 772. , 97–103.

[B61] Olsen S. R. Cole C. V. Watanabe F. S. Dean L. A. (1954). Estimation of Available Phosphorus in Soils by Extraction with Sodium Bicarbonate (Washington, D.C: USDA Circular No. 939, U.S. Government Printing Office).

[B62] Pathak R. K. Pandey D. Pandey V. S. (1977). Stomatal distribution as an index for predicting vigour of plum rootstocks. Indian J. Hortic. 34, 117–119.

[B63] Perez A. Maldonado C. A. Soto I. R. Lopez J. (1988). Dwarfing effect ofinterstems on growth and yield components of mango. J. Agri UnivPuerto Rico 72, 501–508.

[B64] Perez E. G. Roman A. B. Resendiz C. A. Toledano L. M. Vazquez R. M. (1993). Vegetative growth analysis of mango ‘Manila’ trees grafted onto severalinterstock/rootstock combinations. Acta Hortic., 256–263.

[B65] Ravishankar K. V. Chandrashekara P. Sreedhara S. A. Dinesh M. R. Anand L. Saiprasad G. V. S. (2004). Diverse genetic bases of Indian polyembryonic andmonoembryonic mango (*Mangiferaindica*L.) cultivars. Curr. Sci. 87, 870–871.

[B66] Reddy Y. T. N. Raj A. V. V. (2015). Standardization of rootstock in mango. ActaHortic 1066, 99–108. doi: 10.17660/ActaHortic.2015.1066.10

[B67] Reig G. Font Forcada C. Mestre L. (2018). Horticultural, leaf mineral and fruit quality traits of two ‘greengage’ plum cultivars budded on plum based rootstocks in Mediterranean conditions. Sci. Hortic. 232, 84–91. doi: 10.1016/j.scienta.2017.12.052

[B68] Rosa M. R. Juan M. R. Romero L. (2003). Role of grafting in horticultural plants under stress conditions. Food Agric. Environ. 1, 70–74.

[B69] Rossdeutsch L. Schreiner R. P. Skinkis P. A. Deluc L. (2020). Nitrate uptake and transport properties of two grapevine rootstocks with varying vigour. Front. Plant Sci. 11, 2266., PMID: 33537044 10.3389/fpls.2020.608813 PMC7847936

[B70] Sadasivam S. Manickam A. (1992). Biochemical methods for agricultural science (New Delhi: Wiley Easter Limited), 11–12.

[B71] Sairam R. K. (1994). Effect of moisture stress on physiological activities of two contrasting wheat genotypes. Indian J. Exp. Biol. 3, 584–593.

[B72] Sampaio V. R. Simao S. (1996). Effectsofinterstockandgraftingheightonthedevelopment andproductionofmango,var.TommyAtkins. ScientiaAgricola 53, 190–193.

[B73] Sampson J. A. (1961). Methodofreplicatingdryormoisturefacesfor examination by light microscopy. Nature 191, 932933. doi: 10.1038/191932a0, PMID: 13745973

[B74] Sarkhosh A. Shahkoomahally S. Asis C. McConchie C. (2021). Influence of rootstocks on scion leaf mineral content in mango tree (*Mangifera indica* L.). Horticult. Environ. Biotechnol. 62, 725–735. doi: 10.1007/s13580-021-00355-w

[B75] Saroj P. L. Pathak R. K. Yunus M. (1997). Anatomical indices for predicting vigour in clonal rootstocks of guava. Indian J. Hortic. 54, 198–204.

[B76] Satisha J. Prakash G. S. Murti G. S. R. Upreti K. K. (2007). Water stress and rootstocks influences on hormonal status of budded grapevine. Eur. J. Hortic. Sci. 72, 202. doi: 10.1079/ejhs.2007/449536

[B77] Savvas D. Savva A. Ntatsi G. Ropokis A. Karapanos I. Krumbein A.. (2011). Effects of three commercial rootstocks on mineral nutrition, fruit yield, and quality of salinized tomato. J. Plant Nutr. Soil Sci. 174, 154–162. doi: 10.1002/jpln.201000099

[B78] Schechter I. Elfving D. Proctor J. (1991). Apple tree canopy development and photosynthesis as affected by rootstock. Can. J. Bot. 69, 295–300. doi: 10.1139/b91-039

[B79] Shah K. A. Patel M. B. Patel R. J. Parmar P. K. (2010). *Mangiferaindica* (mango). Phcog. Rev. 4, 42–48. doi: 10.4103/0973-7847.65325, PMID: 22228940 PMC3249901

[B80] Singh R. N. Majumder P. K. Sharma D. K. Mukherjee S. K. (1972). Some promising mango hybrids. Acta Hortic. 24, 117–119. doi: 10.17660/ActaHortic.1972.24.20

[B81] Singleton V. L. Orthofer R. Lamuela-Raventos R. M. (1999). Analysis of total phenols and other oxidation substrates and antioxidants by means of Folin-Ciocalteu reagent. Meth. Enzymol. 299, 152–178. doi: 10.1016/S0076-6879(99)99017-1

[B82] Sotiropoulos T. (2006). Performance of the pear (*Pyrus communis*) cultivar William’s Bon Chretien grafted on seven rootstocks. Aust. J. Exp. Agric. 46, 701–705. doi: 10.1071/EA04132

[B83] Sridevi P. Sankaran M. Ravishankar K. V. Shivashankara K. S. Vinay P. Reddy K. (2021). Studies on physiological and biochemical characterization of certain selected half sib populations in mango (*Mangifera indica* L.). Pharma Innovation J. 10, 2327–2333.

[B84] Srivastav M. Kumar M. Dubey A. Singh A. Satram R. K. (2009). Relationship between physiological parameters and vigour indices in polyembryonic genotypes of mango (*Mangifera indica* L.). Indian J. Agric. Sci. 79, 469–471.

[B85] Tworkoski T. Miller S. (2007). Rootstock effect on growth of apple scions with different growth habits. Sci. Hortic. 11, 335–343. doi: 10.1016/j.scienta.2006.10.034

[B86] Uçgun K. Gezgin S. (2017). Can nutritional status of apple trees be determined by leaf analysis in early vegetation? J. Plant Nutr. 40, 277–282.

[B87] USEPA Method 6020B (2014). Inductively Coupled Plasma-Mass Spectrometry (United States Environmental Protection Agency).

[B88] Vahdati K. Sarikhani S. Arab M. M. Leslie C. A. Dandekar A. M. Aleta N.. (2021). Advances in rootstock breedingof nut trees: Objectives and strategies. Plants 10, 2234. doi: 10.3390/plants10112234, PMID: 34834597 PMC8623031

[B89] Valdivia V. V. Garcia S. S. Barraza M. H. P. (2000). Esmeralda interstocks reduce’Ataulfo’mangotreesizewithnoreductioninyield:Resultsoffirstfiveyears. Acta Hortic. 509, 291–296.

[B90] Wang J. Jiang L. Wu R. (2017). Plant grafting: How genetic exchange promotes vascular reconnection. New Phytol. 214, 56–65. doi: 10.1111/nph.14383, PMID: 27991666

[B91] Warschefsky E. J. Klein L. L. Frank M. H. Chitwood D. H. Londo J. P. (2016). Rootstocks: diversity, domestication, and impacts on shoot phenotypes. Trends Plant Sci. 21, 418–437. doi: 10.1016/j.tplants.2015.11.008, PMID: 26698413

[B92] Webster A. D. (2004). Vigour mechanisms in dwarfing rootstocks for temperate fruit trees. Acta Hortic., 29–41. doi: 10.17660/ActaHortic.2004.658.1

[B93] White P. Brown P. (2010). Plant nutrition for sustainable development and global health. Ann. Bot. 105, 1073–1080. doi: 10.1093/aob/mcq085, PMID: 20430785 PMC2887071

[B94] Willis L. E. Marler T. E. (1993). Root and shoot growth patterns of ‘Julie’ and’Keitt’ mango trees. Acta Hortic. 341, 264–270. doi: 10.17660/ActaHortic.1993.341.28

[B95] Xu X. Yu E. Liu L. Zhang W. Wei X. Gao X.. (2013). Dietary intake of vitamins A, C, and E and the risk of colorectal adenoma: a meta-analysis of observational studies. Eur. J. Cancer Prev. 22, 529–539. doi: 10.1097/CEJ.0b013e328364f1eb, PMID: 24064545

[B96] Yahia E. M. Ornelas-Paz J. J. Brecht J. K. García-Solís P. Celis M. E. M. (2023). The contribution of mango fruit (*Mangifera indica* L.) to human nutrition and health. Arabian J. Chem. 16.

[B97] Yonemoto Y. Ogata T. Kozai N. Chusri O. Higuchi H. (2007). Potential of’Khom’ for use as an interstock for compact tree size in mango. Jpn. J. Trop. Agr. 51, 66–69.

[B98] Yong Z. W. Korcak R. F. Miklos F. (1983). Interstock effects on growth,photosynthesis and mineral nutrition of ‘Delicious’ apple seedlings. J. PlantNutr. 6, 597–609.

[B99] Zamboni M. Garavani A. Gatti M. (2016). Vegetative, physiological and nutritional behavior of new grapevine rootstocks in response to different nitrogen supply. Sci. Hortic. 202, 99–106. doi: 10.1016/j.scienta.2016.02.032

[B100] Zapata-Londoño M. B. Ramos-Polo A. Alzate-Arbeláez A. F. Restrepo-Betancur L. F. Rojano B. A. Maldonado-Celis M. E. (2020). Effect of mango (*Mangifera indica*) cv. Azucar juice consumption on plasma and oxidative stress biomarkers. Vitae 27, (1).

[B101] Zarrouk O. Gogorcena Y. Gomez-Aparisi J. (2005). Influence of almond× peach hybrids rootstocks on flower and leaf mineral concentration, yield and vigour of two peach cultivars. Sci. Hortic. 106, 502–514. doi: 10.1016/j.scienta.2005.04.011

[B102] Zekri M. Parsons L. R. (1992). Salinity tolerance of citrus rootstocks: effects of salt on root and leaf mineral concentrations. Plant Soil 147, 171–181. doi: 10.1007/BF00029069

[B103] Zeng H. Wang G. Hu X. (2014). Role of microRNAs in plant responses to nutrient stress. Plant Soil 374, 1005–1021. doi: 10.1007/s11104-013-1907-6

[B104] Zhou Y. Hayat F. Yao J. (2021). Size-controlling interstocks affect growth vigour by down regulating photosynthesis in eight-year old ‘red Fuji’ apple trees. Eur. J. Hortic. Sci. 86, 146–155. doi: 10.17660/eJHS.2021/86.2.5

[B105] Zhou Y. Tian X. Yao J. Zhang Z. Wang Y. Zhang X. (2020). Morphological and photosynthetic responses differ among eight apple scion-rootstock combinations. Sci. Hortic. 261, 108981. doi: 10.1016/j.scienta.2019.108981

